# Adiponectin affects the migration ability of bone marrow-derived mesenchymal stem cells via the regulation of hypoxia inducible factor 1α

**DOI:** 10.1186/s12964-023-01143-y

**Published:** 2023-06-27

**Authors:** Sujung Soh, Sora Han, Hye In Ka, Se Hwan Mun, Woojung Kim, Gaeun Oh, Young Yang

**Affiliations:** 1 Department of Biological Sciences, Sookmyung Women’s University, Seoul, 04310 Republic of Korea; 2 Research Institute of Women’s Health, Sookmyung Women’s University, Seoul, 04310 Republic of Korea

**Keywords:** BMSCs, Adiponectin, HIF1α, Migration, CD44, EL-4, Aging

## Abstract

**Background:**

Bone marrow (BM) is progressively filled with adipocytes during aging process. Thus, BM adipocytes-derived adiponectin (APN) affects the function of bone marrow-derived mesenchymal stem cells (BMSCs). However, little is known about the effect of APN on migration ability of BMSCs cultured under hypoxic conditions, which is similar to the BM microenvironment.

**Results:**

We found that the population and migration ability of BMSCs from APN KO mice was higher than that of WT mice due to increased stability of hypoxia inducible factor 1α (HIF1α). Stem cell factor (SCF)-activated STAT3 stimulated the induction of HIF1α which further stimulated SCF production, indicating that the SCF/STAT3/HIF1α positive loop was highly activated in the absence of APN. It implies that APN negatively regulated this positive loop by stimulating HIF1α degradation via the inactivation of GSK3β. Furthermore, APN KO BMSCs were highly migratory toward EL-4 lymphoma, and the interaction between CD44 in BMSCs and hyaluronic acid (HA) from EL-4 enhanced the migration of BMSCs. On the other hand, the migrated BMSCs recruited CD8^+^ T cells into the EL-4 tumor tissue, resulting in the retardation of tumor growth. Additionally, gradually increased APN in BM on the aging process affects migration and related functions of BMSCs, thus aged APN KO mice showed more significant suppression of EL-4 growth than young APN KO mice due to higher migration and recruitment of CD8^+^ T cells.

**Conclusion:**

APN deficiency enhances CD44-mediated migration ability of BMSCs in the hypoxic conditions by the SCF/STAT3/HIF1α positive loop and influences the migration ability of BMSCs for a longer time depending on the aging process.

Video Abstract

**Supplementary Information:**

The online version contains supplementary material available at 10.1186/s12964-023-01143-y.

## Background

Adiponectin (APN) is mainly produced by epididymal, subcutaneous, and BM adipose tissues and its expression is highly increased by over 100-fold during adipocyte differentiation [1]. In addition, APN plays a role in diabetes [2], atherosclerosis [3], and inflammation [4]. APN also acts as a growth factor for hematopoietic stem cells (HSCs) [5] and osteogenic differentiation factor for mesenchymal stem cells (MSCs) [6]. Since bone marrow (BM) is progressively filled with adipocytes according to the aging progress [7] and secretion of APN from bone marrow adipocytes (BMAs) is higher than that from white adipose tissue [8], it is conceivable that the increase of APN during aging critically affects the function of HSCs and bone marrow-derived mesenchymal stem cells (BMSCs). Hence, it is tempting to study the unknown roles of APN in cellular components of the BM microenvironment. Because the BM microenvironment is influenced by approximately 1–1.5% of low oxygen tension [9, 10], the oxygen level needs to be considered when functions of heterogeneous cell populations in BM are explored [11]. Hypoxia enhances the proliferation [12], stemness [13], and migration [14] of BMSCs. As a molecular mechanism of hypoxia-induced migration, HIF1α/Ca^2+^/NO/ROS axis triggers the migration of BMSCs to recover the hypoxic bone fracture injury site [15].

The tumor microenvironment (TME) consists of a heterogeneous cell population, including immune cells, fibroblast cells, and MSCs, which affects the survival, proliferation, and metastasis of tumor cells [16, 17]. Recent evidence shows that BMSCs can migrate into specific types of tumors in which they become resident MSCs [18–21]. Resident MSCs perform dual roles depending on the tumor context. Many studies have revealed the Janus function of MSCs, which restrains cancer growth or promotes tumor progression and metastasis [22, 23]. Previous studies have shown that BMSCs migrate to tumor sites in response to chemokines secreted from tumors [24] and various inflammatory cytokines, chemokines, and growth factors from cells in the TME [25, 26]. Stromal cell-derived factor (SDF)-1/CXCR4 [27, 28] are well-known ligand and receptor pairs for the recruitment of BMSCs into tumor sites. However, it is not well understood how APN affects the migration of BMSCs from the bone marrow to the tumor sites.

In this study, we showed that APN deficiency stimulated the migration of BMSCs via activation of the SCF/STAT3/HIF1α pathway, and APN treatment negatively regulated this positive loop by stimulating HIF1α degradation via inactivation of GSK3β. Furthermore, BMSCs from APN knockout (KO) mice migrated toward EL-4 lymphoma, and the interaction between CD44 in BMSCs and hyaluronic acid (HA) from EL-4 was responsible for the enhanced migration of BMSCs. Migrated BMSCs secreted CCL8 to recruit CD8^+^ T cells, which suppressed the growth of EL-4 lymphoma. Additionally, aged APN KO mice showed higher suppression of EL-4 growth than young APN KO mice due to the increased migration of BMSCs and recruitment of CD8^+^ T cells compared with young APN KO mice.

## Materials and methods

### Animals

Adiponectin knockout (APN KO) mice were purchased from the Jackson Laboratory on a C57BL/6J background. For all the experiments, young BMSCs and old BMSCs were isolated from 3 to 4-month-old and 11 to 12-month-old APN KO mice, respectively. Age-matched C57BL/6J mice were used as WT controls. Mouse experiments, including the plan and protocols, were conducted according to the guidelines of the Institutional Animal Care and Use Committee (IACUC) after approval by the Institutional Ethical Committee of Sookmyung Women’s University, Seoul, Korea.

### Colony-forming unit-fibroblast (CFU-F) assay

BMSCs were plated into a six well culture plate (SPL) at 1000 cells/well density in Mesencult™ Media (Stem Cell Technologies) at 37 °C under 1% hypoxic conditions and humidity. Half of the medium was changed every three days. After two weeks of culture, the cells were fixed in 10% paraformaldehyde (Sigma) at room temperature for 30 min and stained with 1% crystal violet (Sigma) for 10 min at room temperature. The average number of colonies was counted.

### Bone Marrow-derived Mesenchymal Stem Cell (BMSCs) isolation and culture

BMSCs were obtained from the bone marrow of femurs and tibias of APN KO or C57BL/6J (B6) mice. At least 3-to 4-month-old APN KO or B6 mice were used for young BMSCs, and 11 to 12-month-old APN KO or B6 mice were used for old BMSCs. Specifically, whole bone marrow from the femur and tibia was flushed with 1X phosphate-buffered saline (PBS) with penicillin–streptomycin (Sigma) and passed through a 70 µm of cell strainer (VWR), and 1X RBCs lysis buffer (BioLegend) was used to remove erythrocytes from the BM. Cells were plated in Mesencult™ Media with 1% L-Glutamine (Sigma) and penicillin–streptomycin (Sigma), incubated in a 10% CO_2_, 1% O_2_ hypoxia chamber until 80% confluence, then cells were sub-cultured 1:2 with half media, and all experiments were performed using cells at passage two or three. Moreover, the phenotype of BMSCs was characterized using a flow cytometry cell detachment solution (BioLegend).

### EL-4 bearing in vivo

2 × 10^5^ EL-4 cells were suspended in 0.1 ml 1X PBS (Intron). The cells were subcutaneously injected into mice as previously described [29], and tumor growth was monitored by measuring the tumor volumes (0.5 × (length × width^2^)) with calipers. After 3 weeks of injection, EL-4 lymphoma cells were extracted, weighed, and then digested into single cells. Three independent experiments were performed with at least three mice per group. Single cells were harvested using a 70 μm nylon cell strainer (VWR) and analyzed using flow cytometry (FACS CantoII) at the Core Facility Center for Chronic and Metabolic Diseases at Sookmyung Women’s University.

### RT-PCR and real-time PCR analysis

Total RNA was prepared from BMSCs using RNAiso Plus (TaKaRa), following the manufacturer's protocol. After spectrophotometric quantification (BioTeK), the prepared total RNAs (2 µg) was reverse-transcribed using RevertAid Reverse Transcriptase (Thermo Fisher Scientific) at 37 °C for 1 h. Standard PCR analysis was performed to amplify the mRNAs that encoded the following genes with the primers listed below for 30 PCR cycles with AccuPower PCR PreMix (Bioneer). Each amplification cycle consisted of 10 s denaturation at 94 °C, 15 s annealing at 55 °C, and 20 s extension at 72 °C. Mouse β-actin was used as the control, and the PCR products were electrophoresed on a 1.5% agarose gel stained with RedSafe (Intron).

Real-time PCR analysis was performed to quantify the mRNAs that encoded the following genes with the primers below using SYBR green real-time PCR (SMOBio). Mouse *Gapdh* was used to normalize the results, and the levels of target genes were calculated based on the 2 ^−ΔΔCT^ method using a Quantstudio 3 real-time PCR system (Applied Biosystems). RQ stands for relative quantification. The primer sequences for RT-PCR and real-time PCR are provided in Additional file 2: Table S1 and Table S2.

### Western blotting

For western blotting, cells were directly lysed in Laemmli buffer (50 mM Tris–HCl (pH 6.8), 4% SDS, 10% glycerol, and 5% β-mercaptoethanol) supplemented with a protease inhibitor cocktail (Sigma) and phosphatase inhibitor mixture (Roche). The total protein lysates were incubated on ice for 10 min and then heated at 99 °C for 10 min. The cell supernatants were obtained by centrifugation at 13,500 rpm for 15 min at 4 °C. Proteins were separated electrophoretically on a 10% or 15% SDS–PAGE and transferred onto a 0.45μm pore size nitrocellulose membrane (Cytiva). The membrane was incubated for 18 h with antibodies in 3% BSA (Hanlab) in TBS‐T (150 mM NaCl, 20 mM Tris–HCl (pH 8.0), and 0.05% Tween‐20) at 4 °C, followed by incubation with HRP‐conjugated goat anti-mouse or anti-rabbit IgG (Enzo Life Sciences) in 5% skim milk (Biopure) in TBS‐T at room temperature for 2 h. Proteins were visualized with ECL western blotting reagent using a Fusion Solo‐S image analyzer (Vilber). All images were quantified using ImageJ software.

### Transwell migration assay

A transwell assay was used to analyze the migration ability of BMSCs. Briefly, 5 × 10^4^ BMSCs were placed in the upper chamber of a 24-well transwell plate (VWR) with or without rAPN (10 μg/ml), vitexin (20 μM), and α-CD44 (BioXcell). For co-culture with EL-4, BMSCs and EL-4 were seeded in the upper and bottom chambers, respectively, with or without 4-Mu (2 mM) at a 1:10 ratio (BMSCs: EL-4). After co-culturing for 24 h, the upper chambers were fixed with 4% formaldehyde for 30 min at room temperature (RT). The cells were then stained with 1% crystal violet (Sigma-Aldrich) for 15 min at RT. The cells from the upper surface of the filter membrane were wiped with a cotton swab. Migration ability was evaluated by observing migration to the bottom surface under an optical microscope (Nikon, Tokyo, Japan). Three independent experiments were performed, and the stained areas were analyzed using the ImageJ software.

For the co-culture with splenocytes, BMSCs and splenocytes were seeded in the bottom chamber and the upper chamber, respectively, at 1:10 (BMSCs: splenocytes). Mouse CCL8 chemokine (Peprotech) was treated in the bottom chamber. Before the co-culture with splenocytes, transfection with 25 nM of CCL8 siRNA (Bioneer) on BMSCs was performed using TransIT-TKO® transfection reagent (Mirus) for 24 h. After the co-culture for 18 h, migrated cells into bottom chamber were collected and stained with fluorochrome-conjugated mouse antibodies (Biolegend). The CCL8 siRNA sequences used in this experiment are provided in Additional file 2: Table S4.

### Preparation of cells

Spleens, tibias, and femurs were isolated from mice after incubation, and single cells were harvested with a 70μm nylon cell strainer (VWR) after the residual erythrocytes had been lysed with 1X RBC lysis buffer (BioLegend). Solid tumors were chopped into small pieces and passed through 100-μm nylon cell strainers (VWR), which were then harvested and centrifuged at 13,500 rpm for 15 min at 4 °C. The viability and number of isolated cells from individual tissues were determined using trypan blue exclusion counts.

### Flow cytometry analysis

The following antibodies were used for the flow cytometry analysis: FITC-, PE-, PECy7-, APC- and PerCP5.5-conjugated anti-mouse antibodies: anti-CD45, anti-CD11b, anti-Sca-1, anti-CD44, anti-CD3, anti-CD8, anti-CD29, and anti-c-kit (BioLegend). The cells were incubated with fluorochrome-conjugated mouse Abs directed against the cell surface at 4 °C for 30 min. The cells were washed with 1X PBS and resuspended in a 10% formalin solution (Sigma). The labeled cells were analyzed on a FACS Canto II cytometer equipped with FACS Diva software (BD Biosciences), and the flow cytometry data were analyzed using FlowJo software (Tree Star).

### CHIP-qPCR

Chromatin immunoprecipitation (CHIP) was conducted according to the previous report [30]. CHIP was performed on 2 × 10^6^ BMSCs from WT and APN KO mice with a CHIP assay kit (Millipore), according to the manufacturer’s instructions, with an anti-pSTAT3 (Cell Signaling Technology) or rabbit IgG (Sigma) as the negative control in triplicates. The final libraries were quantitated with SYBR green real-time PCR (SMOBio). Putative pSTAT3 binding site in the HIF1α promoter were identified using JASPAR3 program (https://jaspar.genereg.net/matrix/MA0144.1/). Results were analyzed as fold enrichment relative to the IgG control. The primer sequences for CHIIP-qPCR are provided in Additional file 2: Table S3.

### Immunohistochemistry

Immunohistochemistry (IHC) assay was performed according to the protocol published [31, 32]. All EL-4 tumor tissues were sectioned into 5 μm-thick slides. The slides were deparaffinized in two times of xylene, two times of 100% ethanol, and followed by a serial of diluted ethanol (95% and 75%) for 5 min each. Freshly made citrate buffer pH.6.0 (Sigma) was used for the antigen retrieval step. After permeabilizing the samples with 0.1% Triton X 100 for 10 min, the slides were blocked for 1 h at RT with 5% blocking reagent (5% IgG Free BSA + 5% filtered FBS (Gibco) in 1 X PBS (Intron)). The primary antibodies were diluted in 1% blocking reagent and incubated for overnight at 4℃. All staining included an isotype control for the negative control. Then the slides were washed PBS-T (1X PBS + 0.1% Tween-20 (Duchefa)) for five times at 4℃ incubated secondary antibodies for 45 min at RT. The images were visualized with Cytation 5 (BioTek).

### Production and purification of recombinant APN

Construct of pET28a-His tagged APN (without signal peptide region) was transformed into *Escherichia coli* strain BL21 cells, and recombinant APN protein (rAPN) was induced with 0.1 mM isopropyl-beta-d-thiogalactopyranoside (IPTG) treatment overnight at 37 °C. Cells were harvested by centrifugation at 6000 rpm for 20 min at 4 °C, resuspended in lysis buffer (50 mM NaH_2_PO_4_, 300 mM NaCl, 10 mM imidazole, 1 mM DTT 1 mM PMSF, 1% Triton X-100, and 5% glycerol, pH 8.0), and sonicated with a pulse rate of 30 s on and 30 s off for 20 min. After centrifugation at 13,500 rpm for 15 min at 4 °C, the supernatant was collected in a 15 ml tube. According to the manufacturer’s instructions, rAPN was purified using a HisTrap column (Cytiva) and BioRad equipment (BioLogic™ Low-pressure Liquid chromatography systems). The eluted rAPN was dialyzed with a dialysis cassette (Thermo Scientific) in dialysis buffer (50 mM Tris–HCL (pH 8.0), 1 mM EDTA, 10% Glycerol, 150 mM NaCl, (pH 8.0)) at 4 °C overnight. The rAPN was subsequently concentrated using centrifugal filter columns (Millipore). The concentration of rAPN was determined using the Albumin Standard (Thermo Scientific), and the purity and expression of rAPN were confirmed using SDS-PAGE and western blotting. For the recovery experiments, 10 μg/ml of rAPN was added for 24 h.

### Chemokine panel (CCL8) and ELISA (APN and CCL8)

BMSCs from WT and APN KO mice were cultured in Mesencult^TM^ Media (Stem Cell Technologies) until they reached 90% confluence. Samples of each BMSCs lysate were analyzed for specific proteins using proteome profiler arrays, specifically the Proteome Profiler™ Array (R&D Systems, ARY020), according to the manufacturer’s instructions. Quantifying the detected spots was performed using a Fusion Solo‐S image analyzer (Vilber) and the dots were analyzed using ImageJ software. For the APN ELISA, the lysate of BM and WAT was obtained from WT and APN KO mice, and then the assay was performed using the Mouse Adiponectin/Acrp30 ELISA kit (R&D Systems), according to the manufacturer’s protocol. For the CCL8 ELISA, the same amount of BMSCs was seeded in 6 well, and the cell culture supernatant from BMSCs was collected after 24 h. The ELISA assay was performed using Mouse CCL8 ELISA Kit (Abcam), according to the manufacturer’s protocol.

### Antibodies and chemicals

The following antibodies and chemicals were used: HIF1α, STAT3, pSTAT3 (Y705), pGSK3β (S9), pAKT (S473), AKT, GSK3β, RhoA, Rock1, pMLC2 (T18/S19), MLC2, and MYLK (Cell Signaling Technology), β-actin (Santa Cruz Biotechnology), HIF1α inhibitor, vitexin (Sigma), GSK3β inhibitor, SB216763 (Sigma), c-KIT inhibitor, DCC-2618 (Sigma), STAT3 inhibitor, stattic (Sigma), HASs inhibitor, 4-Methylumbelliferone (Sigma). For IHC staining, CD8 (BioLegned), HIF1α (Cell Signaling Technology), CD44 (BD Biosciences) and Sca-1 (Invitrogen) were used for primary antibodies, and anti-rat FITC (Sigma), anti-rabbit Alexa Fluor 594 (Invitrogen) were used for secondary antibodies.

### Statistical analysis

All data were presented as the mean ± standard deviation (SD) of at least three independent experiments. GraphPad software 5.0 was used for the statistical analysis. Comparisons were analyzed using Student’s *t*-test and one-way analysis of variance (ANOVA). The FACS data are representative of at least three separate experiments. Statistical significance was set at *P*-value < 0.05. * Represented a *P*-value < 0.05, ** represented a *P*-value < 0.01, *** represented a *P*-value < 0.001.

## Results

### APN deficiency increases the stemness, proliferation, and migration of BMSCs

**Fig. 1:**
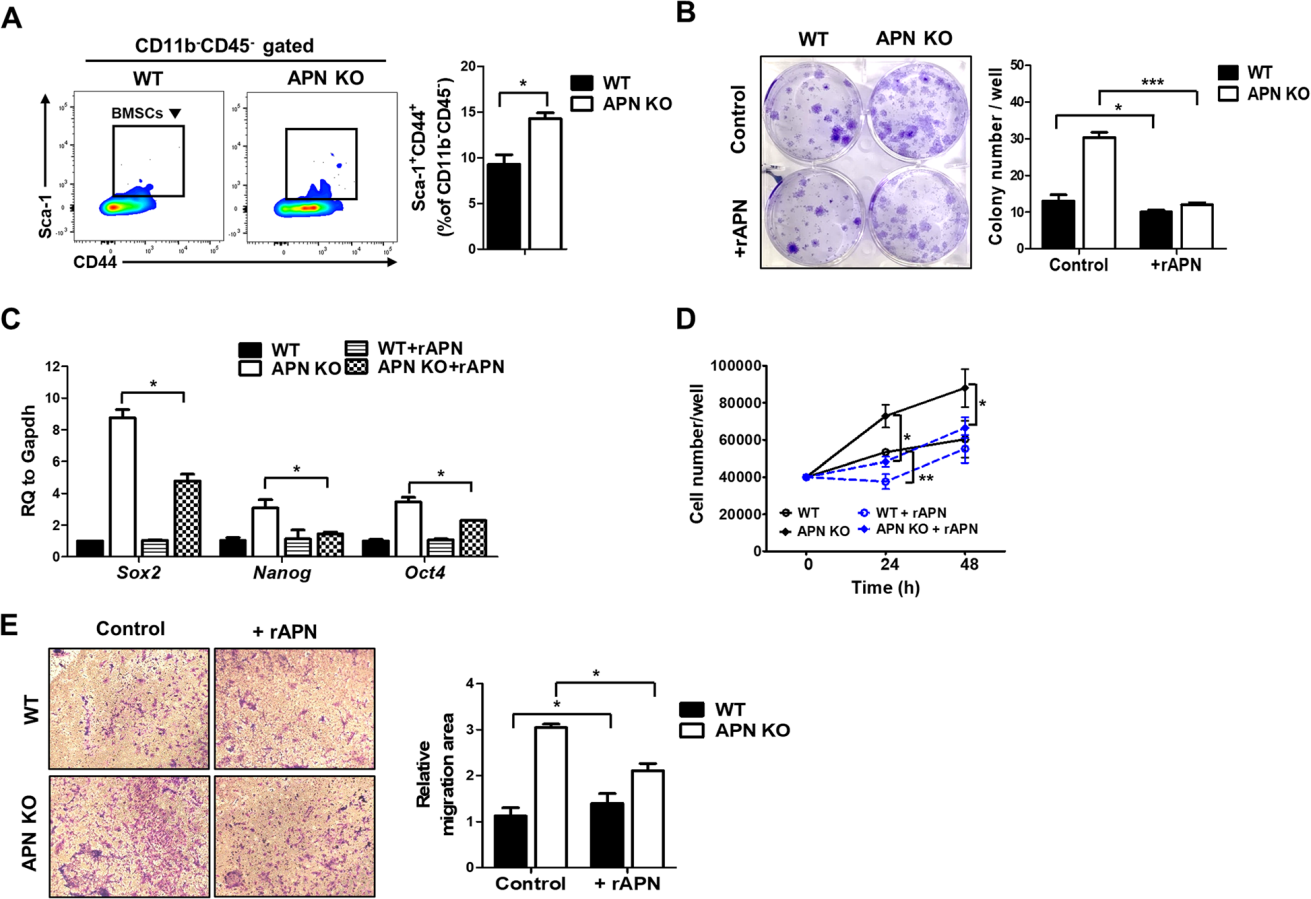
Deficiency of APN increases the stemness, proliferation, and migration of BMSCs. **A** The population of BMSCs (Sca-1^+^CD44^+^CD11b^−^CD45^−^) cultured under 1% hypoxia was analyzed by flow cytometry, and bar graphs were used for quantitative data. **B** APN KO BMSCs were seeded and colonies were stained after 14 days. The graph showed the number of colonies stained per well. **C** The mRNA levels of *Sox2*, *Nanog*, and *Oct4* were measured using real-time PCR. **D** APN KO BMSCs were cultured in the presence or absence of rAPN and cell numbers were measured at the indicated times. **E** APN KO BMSCs were placed in the upper chamber of the transwell plate and migrated cells were imaged under a light microscopy (× 100 magnification) after 24 h, and then images were quantified using ImageJ software. All data were expressed as the mean ± SD from at least three independent experiments. *p < 0.05, **p < 0.01, ***p < 0.001

BMSCs were cultured under 1% hypoxic conditions, and the population of Sca-1^+^ CD44^+^ CD11b^−^ CD45^−^ BMSCs was measured at passage two. BMSCs from APN KO mice were more enriched compared to WT BMSCs (Fig. 1A). The functional difference between APN KO and WT BMSCs was determined. Firstly, the self-renewability of BMSCs was examined using a colony-forming unit-fibroblast (CFU-F) assay. APN KO BMSCs showed a two-fold increase in colony-forming ability compared with WT BMSCs (Fig. 1B). Surprisingly, markers of pluripotency and self-renewability, including *Sox2*, *Nanog*, and *Oct4* mRNA, were enhanced in APN KO BMSCs, and treatment with recombinant APN (rAPN) slightly reduced these levels (Fig. 1C). Secondly, proliferation rates of the WT and APN KO BMSCs were compared. APN KO BMSCs showed a higher proliferation rate than WT BMSCs, and rAPN treatment reduced the proliferation of APN KO BMSCs (Fig. 1D). Lastly, because BMSCs can move from their niche into target tissues by passing through vessel walls, the mobility of BMSCs was examined. The migratory potential of APN KO BMSCs was enhanced and reduced by rAPN treatment (Fig. 1E).

### HIF1α expression in APN KO BMSCs is associated with activation of STAT3

**Fig. 2:**
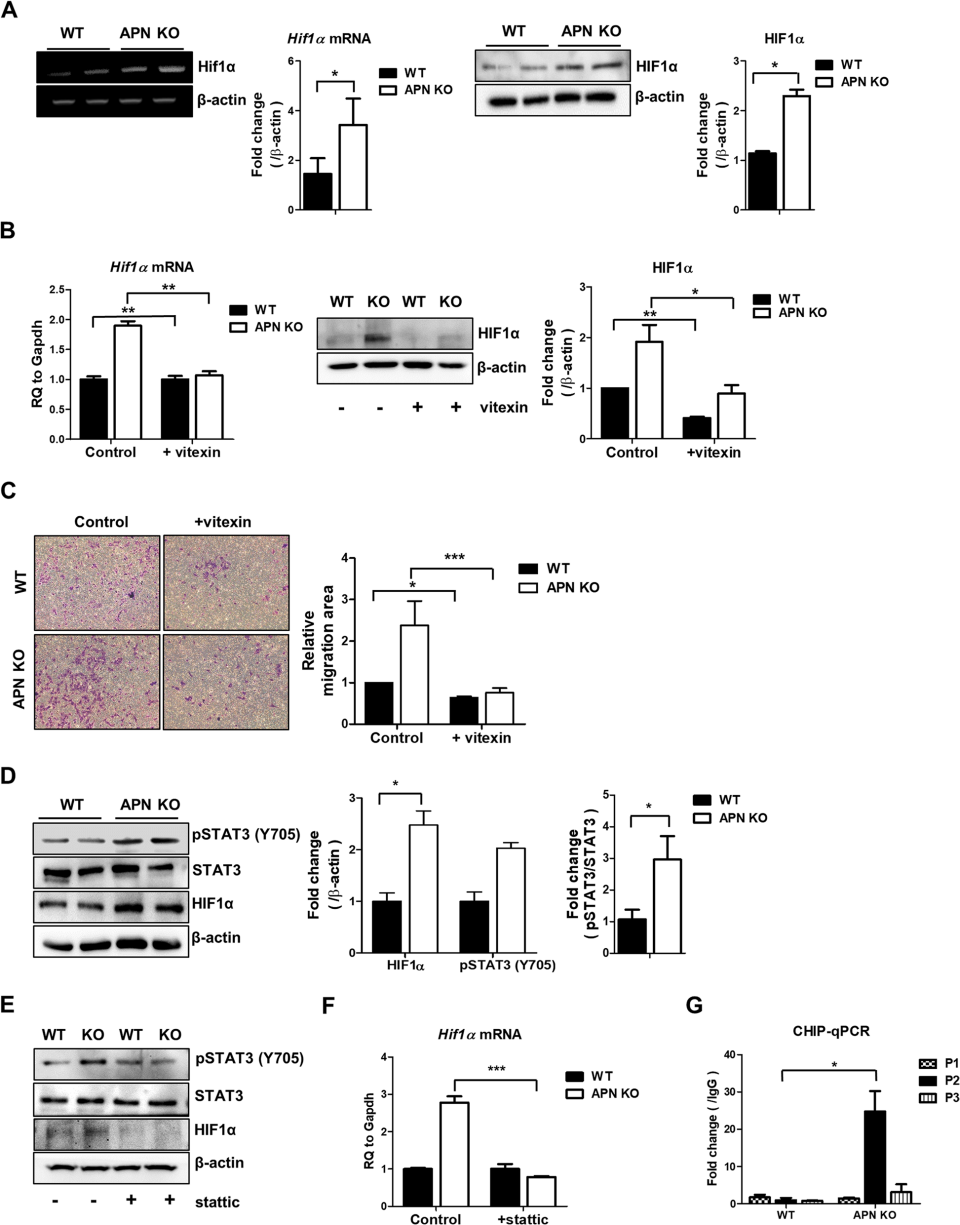
STAT3 activation is responsible for HIF1α increase in APN KO BMSCs. **A** The mRNA and protein levels of HIF1α were measured by RT-PCR and western blotting, respectively, and bar graphs were used for quantitative data. **B** APN KO BMSCs were cultured in the presence or absence of vitexin (20 μM). The mRNA and protein levels of HIF1α were measured using real-time PCR and western blotting, respectively. **C** APN KO BMSCs were placed in the upper chamber of the transwell plate with or without vitexin, and the migrated cells were imaged under light microscopy (× 100 magnification) after 24 h. **D** The protein levels of STAT3, pSTAT3 (Y705), and HIF1α were analyzed by western blotting, and bar graphs were used for quantitative data. **E**,** F** APN KO BMSCs were cultured in the presence or absence of static (5 nM), and the protein levels of STAT3, pSTAT3 (Y705), and HIF1α were analyzed by western blotting, and bar graphs were used for quantitative data. The mRNA levels of *HIF1α* were measured by real-time PCR. **G** CHIP-qPCR was performed to confirm the binding site of pSTAT3 to specific regions of HIF1α promoter (P). All images were quantified using ImageJ software. All data were expressed as the mean ± SD from at least three independent experiments. *p < 0.05, **p < 0.01, ***p < 0.001

As APN KO BMSCs show enhanced migration and the migration of MSCs is mediated by HIF1α [33], the level of HIF1α in APN KO BMSCs was examined. The levels of HIF1α in mRNA and proteins increased in APN KO BMSCs (Fig. 2A). To determine whether the increased HIF1α affects the migration of BMSCs, BMSCs cultured in the upper chamber of transwells were treated with HIF1α inhibitor, vitexin. The treatment of vitexin decreased the mRNA and protein levels of HIF1α (Fig. 2B) and HIF1α inhibition suppressed the increased migration of APN KO BMSCs (Fig. 2C). We next examined whether the STAT3 signaling pathway is associated with increased HIF1α in APN KO BMSCs. Phosphorylation on Y705 of STAT3 was enhanced in APN KO BMSCs compared with that in WT BMSCs (Fig. 2D), and treatment with a STAT3 inhibitor, stattic, reduced the phosphorylation level on Y705 of STAT3. The resulting inhibition of STAT3 suppressed HIF1α mRNA and protein levels in the APN KO BMSCs (Fig. 2E, F). We have performed CHIP-qPCR to prove whether pSTAT3 directly binds to the HIF1α promoter. pSTAT3 binding to HIF1α promoter was increased in APN KO BMSCs compared to WT BMSCs (Fig. 2G and Additional file 1: Figure S1A).

### SCF increases HIF1α expression via STAT3

**Fig. 3:**
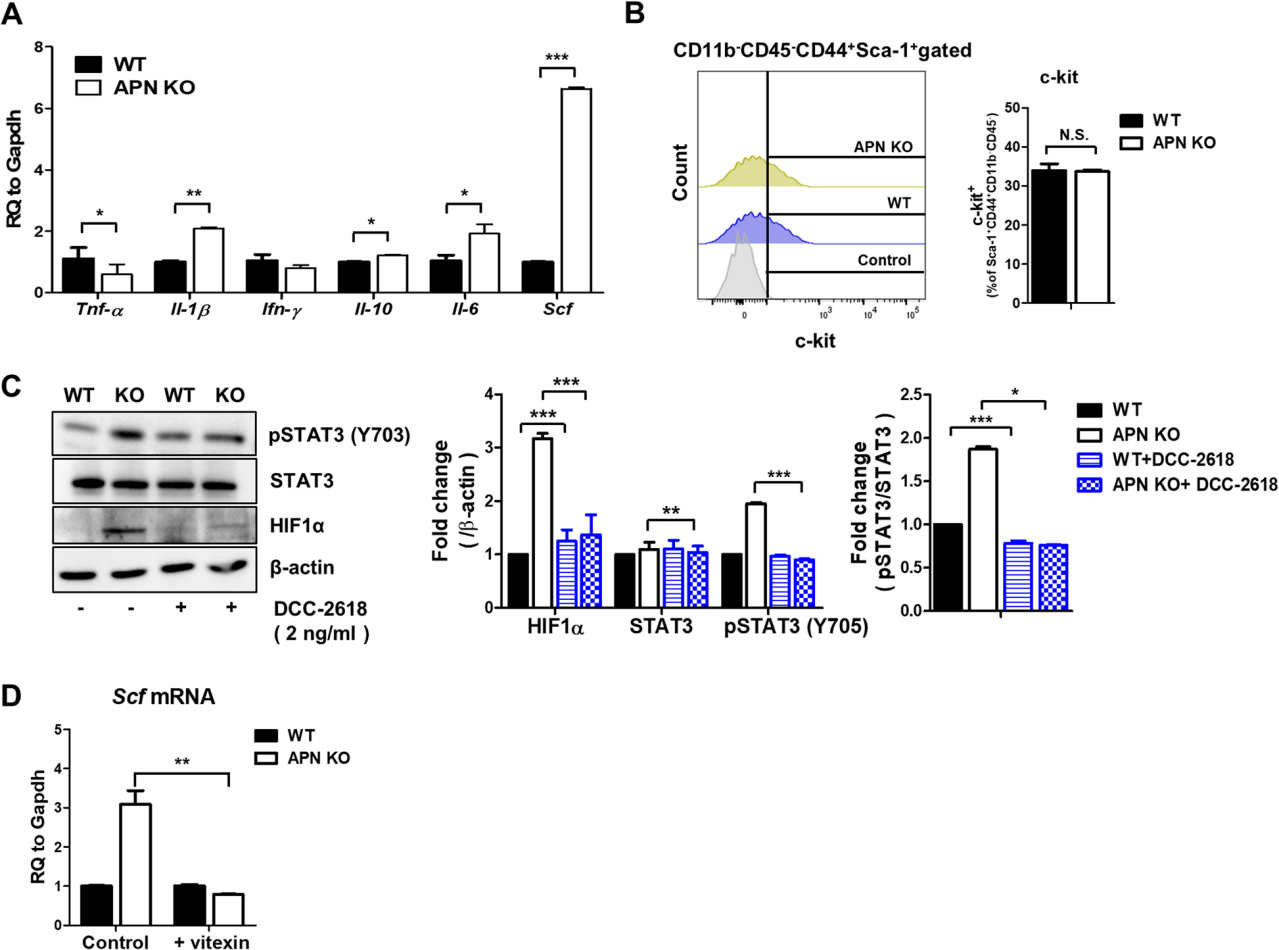
HIF1α stimulates SCF expression. **A** The mRNA levels of *Tnf-α*, *Scf*, *Il-1β, Ifn-γ*, *Il-10*, and *Il-6* were measured by real-time PCR in BM and BMSCs. **B** Levels of c-kit were analyzed using flow cytometry, and bar graphs were used for quantitative data. **C** APN KO BMSCs were cultured in the presence or absence of DCC-2618 (2 ng/ml), and the protein levels of STAT3, pSTAT3 (Y705), and HIF1α were analyzed by western blotting, and bar graphs were used for quantitative data. **D** APN KO BMSCs were cultured in the presence or absence of vitexin (20 μM), and the mRNA level of *Scf* was measured by real-time PCR. N.S stands for not significant. All data were expressed as the mean ± SD from at least three independent experiments. *p < 0.05, **p < 0.01, ***p < 0.001

To determine the reason why APN KO BMSCs show STAT3 activation compared to WT BMSCs, the level of various cytokines activating STAT3 [34] was measured. APN KO BMSCs expressed high level of SCF, while other cytokines showed no significant differences (Fig. 3A). Next, the expression level of c-KIT, the receptor for SCF, was examined, but no difference in c-KIT levels was observed (Fig. 3B). Thus, we determined whether STAT3 activation was inhibited by c-KIT inhibitor, DCC-2618. The treatment of c-KIT inhibitor reduced the activation of STAT3 and expression of HIF1α, indicating that the increase in SCF causes STAT3 activation with no change in its receptor level (Fig. 3C).

Since HIF1α acts as a crucial transcriptional regulator under hypoxic culture conditions, it is possible that the increase in SCF might be regulated by HIF1α. Indeed, treatment with vitexin suppressed the increase in *Scf* mRNA in APN KO BMSCs (Fig. 3D). These results imply that HIF1α and SCF are positively regulated by an autocrine loop.

### APN deficiency induces HIF1α accumulation by GSK3β inhibition

**Fig. 4:**
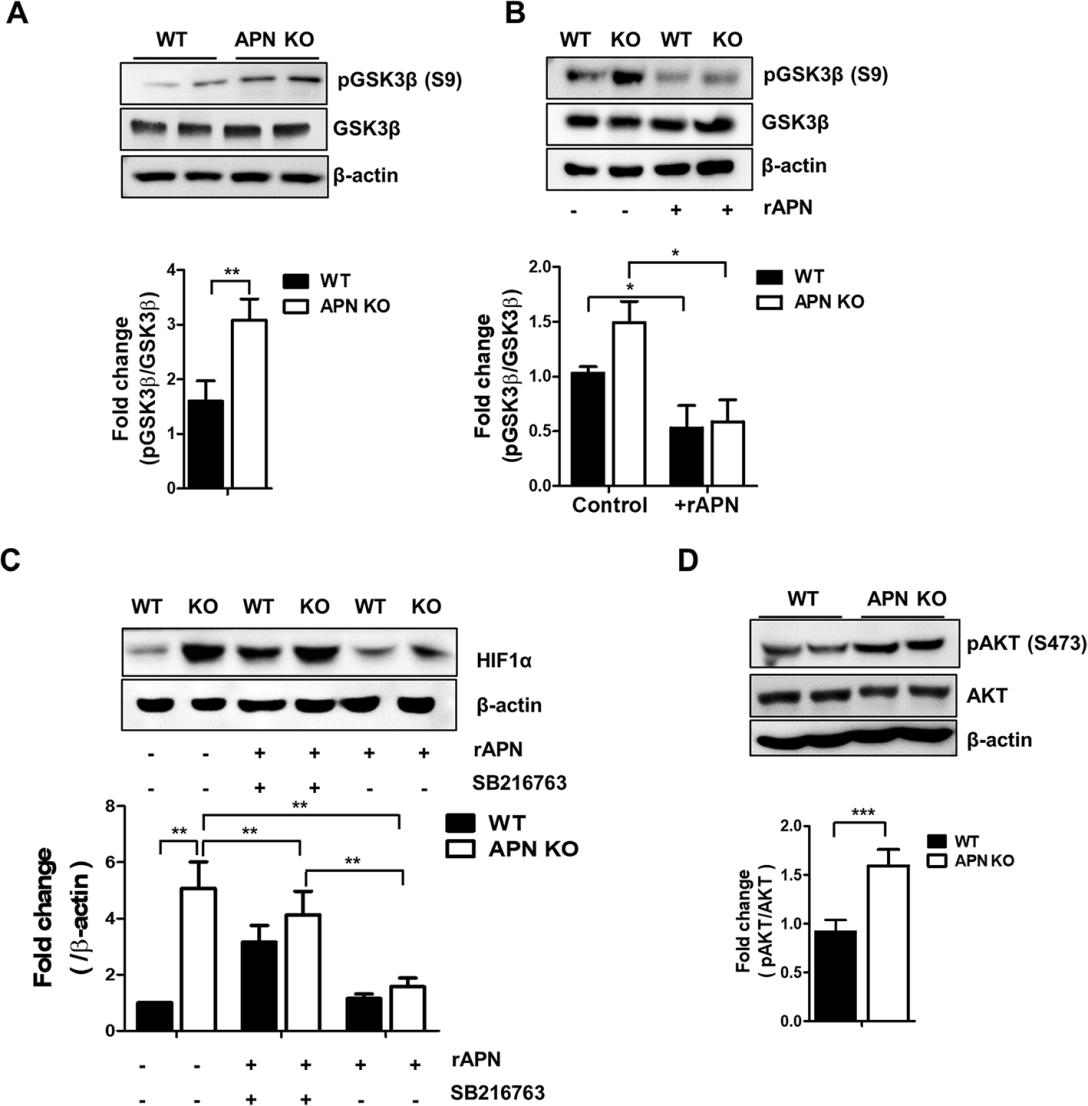
APN-induced GSK3β inactivation inhibits STAT3/HIFα axis. **A** Protein levels of pGSK3β (S9) and GSK3β were analyzed by western blotting. **B** APN KO BMSCs were cultured in the presence or absence of rAPN (10 μg/ml), and the protein levels of pGSK3β (S9) and GSK3β were analyzed by western blotting. **C** APN KO BMSCs were pretreated with SB216763 (10 μM) for 3 h followed by the treatment of rAPN (10 μg/ml) for 24 h, and the protein level of HIF1α was analyzed by western blotting. **D** The protein levels of pAKT (S473), and AKT in APN KO BMSCs were analyzed by western blotting. All images were quantified using ImageJ software, and bar graphs were used for quantitative data representation. All data were expressed as the mean ± SD from at least three independent experiments. *p < 0.05, **p < 0.01, ***p < 0.001

It is known that APN activates GSK-3β by inhibition of phosphorylation on S9 of GSK3β [35] and the active form of GSK3β degrades HIF1α [36]. Therefore, we hypothesized that the absence of APN leads to the accumulation of HIF1α through the inactivation of GSK3β. Indeed, APN KO BMSCs showed an approximately two-fold increase in the phospho-GSK3β (inactive form) without a change in the total level of GSK3β (Fig. 4A), and rAPN treatment decreased the inactive form of GSK3β (Fig. 4B), indicating that the increase of the inactive form of GSK3β contributes to the accumulation of HIF1α in APN KO BMSCs. To further confirm this result, APN KO BMSCs were pretreated with the GSK3β inhibitor, SB216763. The inhibition of GSK3β increased HIF1α expression even following the treatment of rAPN (Fig. 4C). It implies that GSK3β is the crucial downstream regulator of rAPN-induced degradation of HIF1α. As an upper stream regulator of GSK3β, AKT was also activated in APN KO BMSCs (Fig. 4D). Taken together, APN deficiency may inactivate GSK3β through AKT activation, which leads to HIF1α accumulation.

### High expression of CD44 is associated with an increase in migration of APN KO BMSCs

**Fig. 5:**
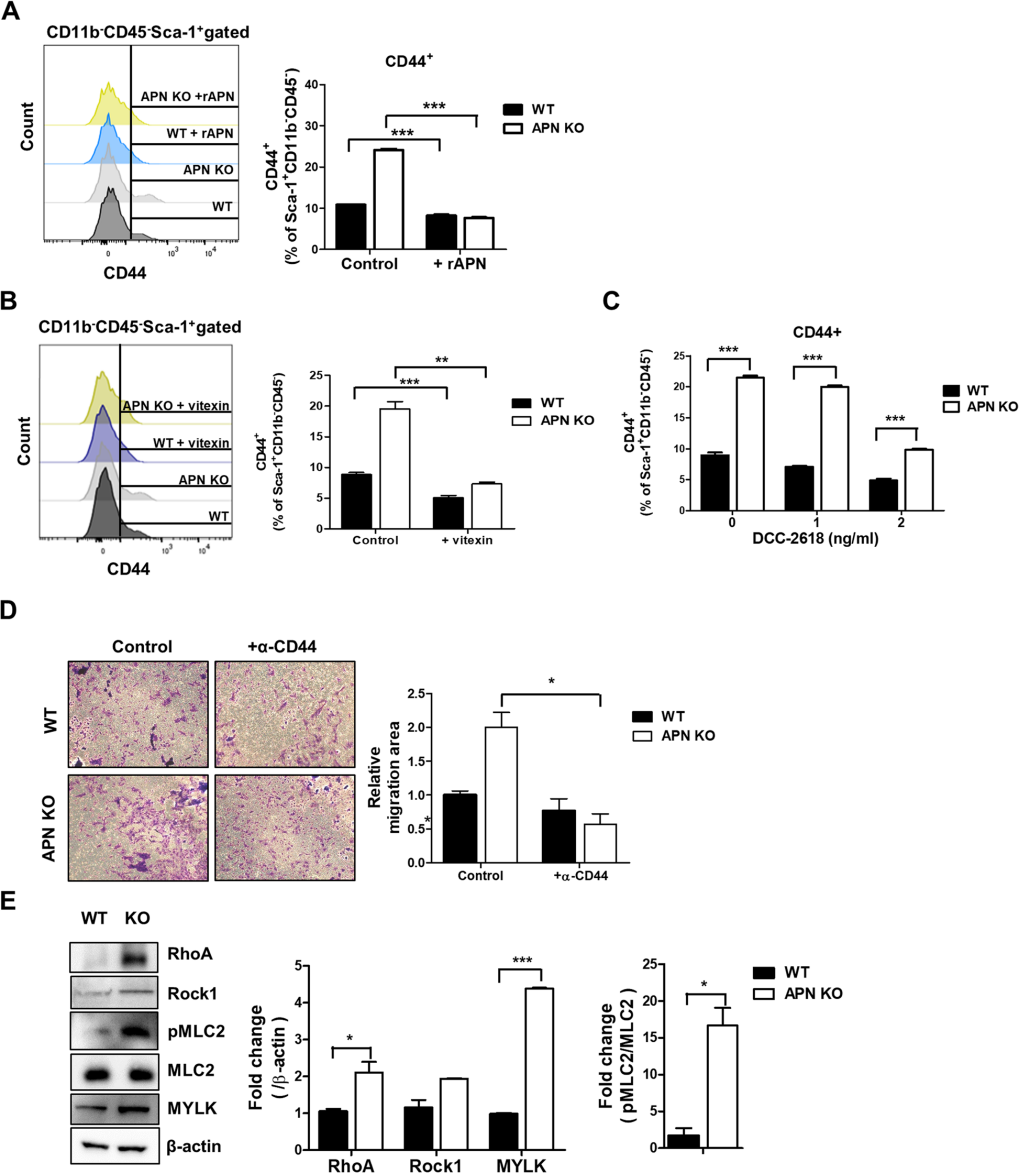
High expression of CD44 is associated with increase in migration of APN KO BMSCs.** A** APN KO BMSCs were cultured in the presence or absence of rAPN (10 μg/ml), and CD44 expression was analyzed by flow cytometry. **B** APN KO BMSCs were cultured in the presence or absence of vitexin (20 μM), and CD44 expression was analyzed by flow cytometry. **C** APN KO BMSCs were cultured with or without DCC-2618, and CD44 expression was analyzed by flow cytometry. **D** APN KO BMSCs were placed in the upper chamber of the transwell plate with or without α-CD44, and migrated cells were imaged under a light microscopy (× 100 magnification) after 24 h. All images were quantified using ImageJ software. **E** Protein levels of RhoA, Rock1, pMLC2 (T18/S19), MLC2, and MYLK were analyzed by western blotting. All images were quantified using ImageJ software, and bar graphs were used for quantitative data. All data were expressed as the mean ± SD from at least three independent experiments. *p < 0.05, **p < 0.01, ***p < 0.001

Subsequently, we focused on how the increase in HIF1α in APN KO BMSCs is linked to the enhanced migration. It is known that CD44 is a migration-related gene [37] and is transcribed by HIF1α [38]. The level of CD44 in APN KO BMSCs was approximately two-fold higher than that of WT BMSCs (Fig. 5A). When APN KO BMSCs were treated with vitexin and inhibitors of c-kit, DCC-2618, the level of CD44 decreased (Fig. 5B, C). It means that the activation of SCF/STAT3/HIF1α axis enhances CD44 expression. The anti-CD44 antibody-mediated inactivation of CD44 signaling pathway was reduced the migration ability of APN KO BMSCs (Fig. 5D). On the other hand, APN KO BMSCs showed increase in levels of RhoA, ROCK1, and pMLC2 (T18/S19) (Fig. 5E), which are associated with the reorganization of the cytoskeleton for mobilization [39]. These results imply that the activation of the SCF/STAT3/HIF1α axis is responsible for the enhanced CD44 expression, which causes increase in migration ability of APN KO BMSCs.

### Binding CD44 and HA is associated with the enhanced migration ability of APN KO BMSCs

**Fig. 6:**
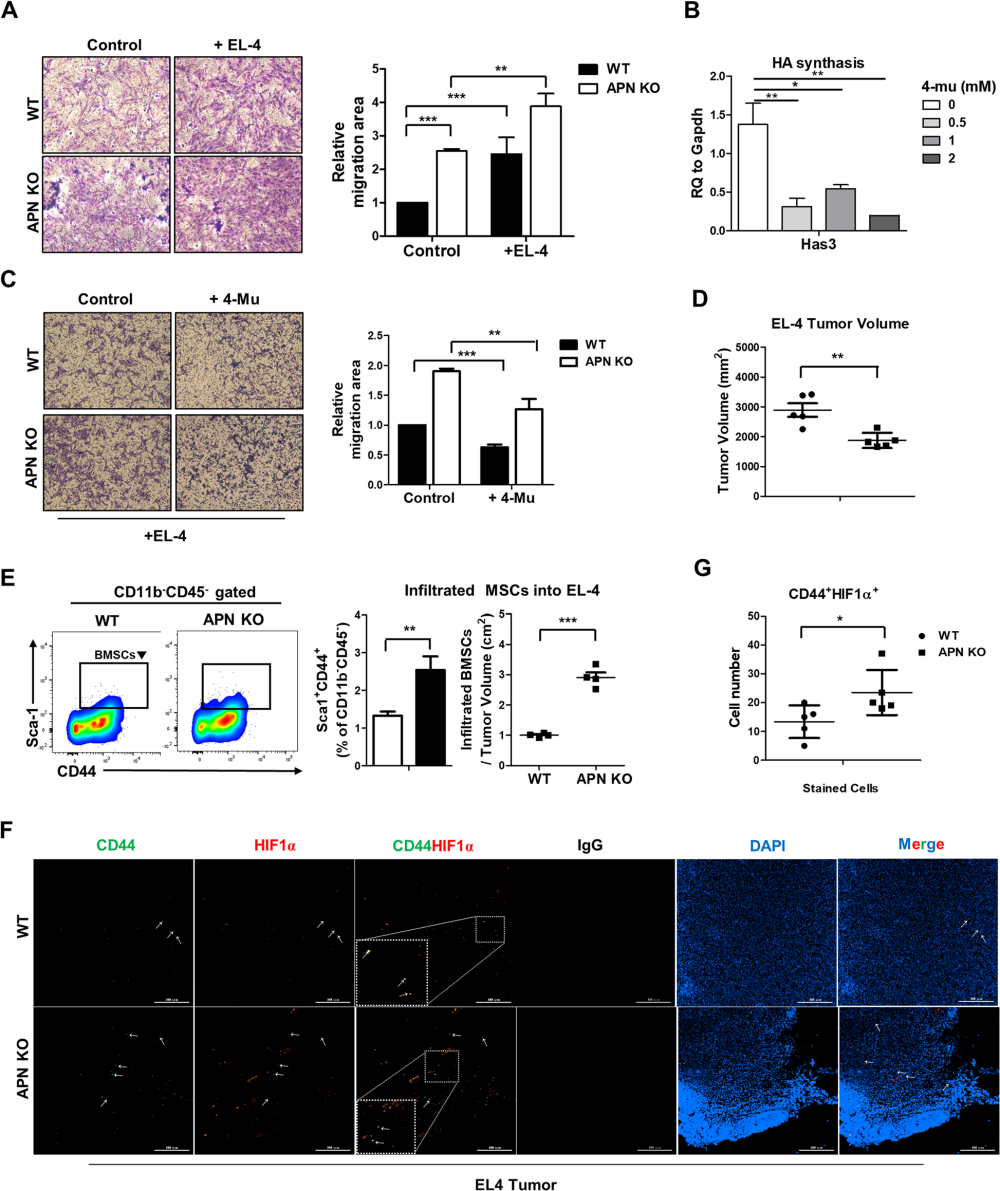
BMSCs within EL-4 tumor stimulates the infiltration of CD8^+^ T cells. **A** APN KO BMSCs and EL-4 cells were placed in the upper and lower chambers of the transwell plate, respectively. Migrated cells were imaged under light microscopy (× 100 magnification) after 24 h. **B** The mRNA level of *Has3* in EL-4 cells was measured by real-time PCR after treatment at the indicated concentration of 4-Mu for 24 h. **C** APN KO BMSCs were placed in the upper chamber of the transwell plate, and EL-4 was placed in the bottom chamber of the transwell plate with or without 4-Mu (2 mM), and migrated cells were imaged under light microscopy (× 100 magnification) after 24 h. **D** EL-4 tumors were excised from WT and APN KO mice 21 days after administration, and tumor volume was measured. **E** The population of infiltrated MSCs was analyzed by flow cytometry. All images were quantified using ImageJ software, and bar graphs were used for quantitative data. **F**,** G** Representative immunofluorescence IHC staining images were used to detect the colocalization of HIF1α and CD44 from EL-4 tumor sections and shown as a quantitative bar graph. Scale bar = 300 μm. All data were expressed as the mean ± SD from at least three independent experiments. *p < 0.05, **p < 0.01, ***p < 0.001

It is well-known that BMSCs move to the injured area to remodel the tissues [40]. Therefore, it prompted us to investigate whether APN KO BMSCs is indeed migrated to other tissues in vivo. To prove this possibility, co-culture with tumor cells and syngeneic tumor models were employed to examine the migration of BMSCs into tumor sites. As binding between CD44 and HA plays a key role in the migration of BMSCs, the hyaluronic acid synthase-3 (HAS3) expression was compared among the various tumor cells. EL-4 tumor cells were highly expressed *Has3* mRNA. Thus, EL-4 tumor cells were selected for the further experiment (Additional file 1: Figure S2A). Firstly, the migration of APN KO BMSCs into tumor sites was determined using a transwell assay, which revealed that the migration of APN KO BMSCs toward EL-4 lymphoma cells was enhanced compared with that of WT BMSCs (Fig. 6A). When EL-4 cells were treated with the HA synthesis inhibitor, 4-Methylumbelliferone (4-Mu), the migration of APN KO BMSCs was significantly reduced (Fig. 6B, C). To further confirm whether APN KO BMSCs migrate toward the EL-4 tumor mass in vivo, a syngeneic tumor model using EL-4 was used. EL-4 tumor mass from APN KO mice showed lower tumor weight and volume than those of WT mice (Fig. 6D), consistent with a previous report [29]. Although the EL-4 tumor mass in APN KO mice was smaller than that in WT mice, tumor-infiltrated BMSCs were two-fold higher in APN KO mice than those in WT mice and the infiltrated APN KO BMSCs maintained HIF1α expression (Fig. 6E-G), indicating that APN KO BMSCs highly migrate to tumor sites.

### CCL8 secreted from APN KO BMSCs recruits CD8^+^ T cells into the tumor mass

**Fig. 7:**
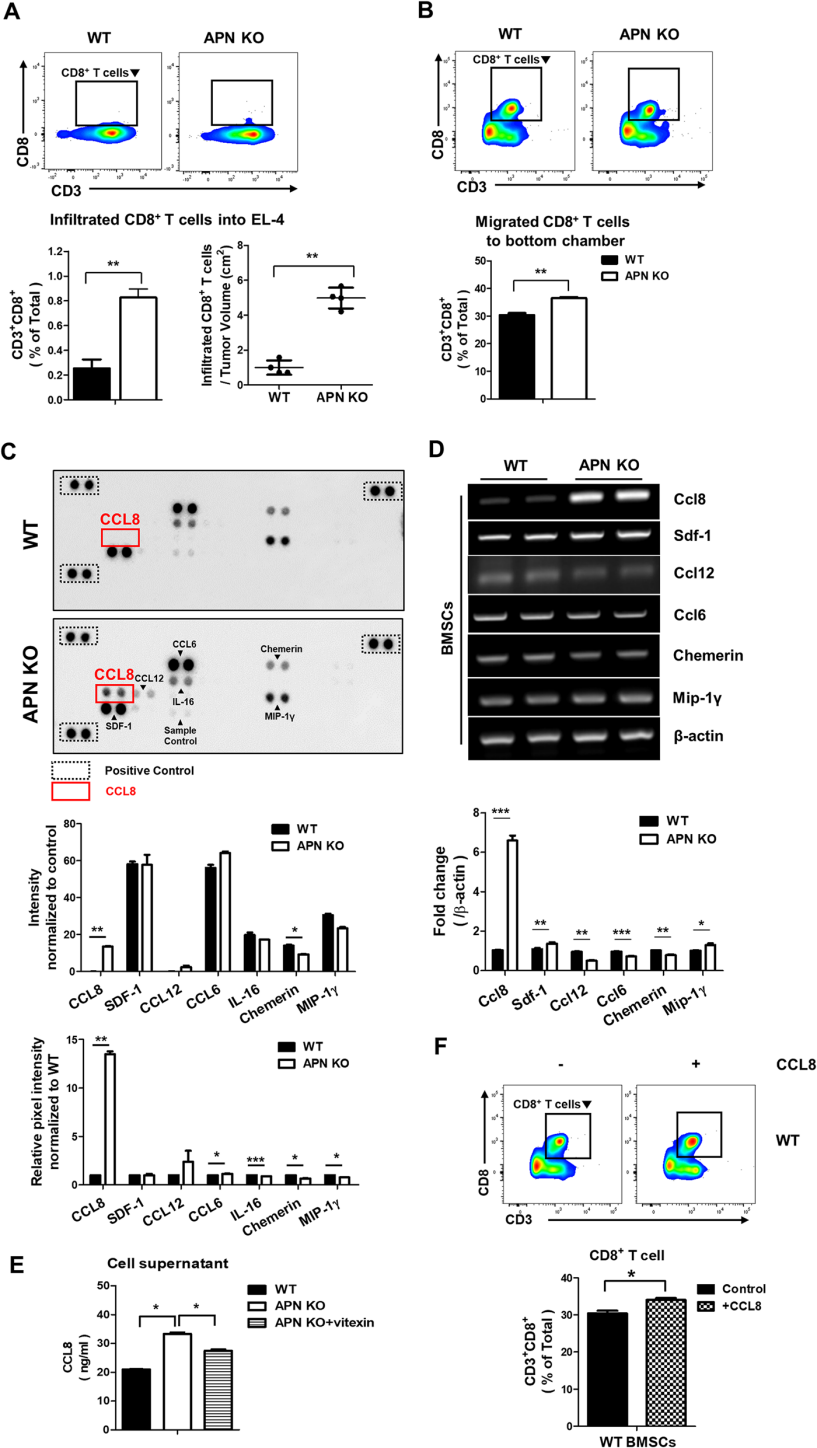
CCL8 secreted from APN KO BMSCs within EL-4 tumor recruits CD8^+^ T cells. **A** The population of infiltrated CD8^+^ T cells was analyzed by flow cytometry. **B** APN KO BMSCs were placed in the bottom chamber, splenocytes from EL-4-bearing WT mice were placed in the upper chamber of the transwell plate, and the population of migrated CD8^+^ T cells in the bottom chamber was analyzed by flow cytometry. **C** Cell lysates from the BMSCs were used to measure the levels of various chemokines. All dots were quantified using ImageJ software, and bar graphs were used for quantitative data. **D** The mRNA levels of the indicated chemokines were measured using RT-PCR and real-time PCR. **E** The level of CCL8 in cell culture supernatant with or without the treatment of vitexin (20 μM) in WT and APN KO BMSCs was measured. **F** WT BMSCs were placed in the bottom chamber of the transwell plate with or without CCL8 (10 ng/ml). Splenocytes from EL-4-bearing WT mice were placed in the upper chamber of the transwell plate and migrated CD8.^+^ T cells in the bottom chamber were analyzed by flow cytometry. All images were quantified using ImageJ software, and bar graphs were used for quantitative data. All data were expressed as the mean ± SD from at least three independent experiments. *p < 0.05, **p < 0.01, ***p < 0.001

To explore the role of the recruited BMSCs in tumor tissues, infiltrated CD8^+^ T cells were examined using flow cytometry. CD8^+^ T cell infiltration was highly enhanced in tumor-bearing APN KO mice (Fig. 7A). The infiltrated CD8^+^ T cells were also observed using IHC (Additional file 1: Figure S3A). In addition, the number of CD8^+^ T cells in the spleen increased in EL-4-bearing APN KO mice (Additional file 1: Figure S3B). To determine whether the migrated BMSCs recruit CD8^+^ T cells, splenocytes isolated from EL-4-bearing mice were cultured in the upper well and BMSCs were cultured in the bottom well, and migrated splenocytes into BMSCs were characterized using the flow cytometry analysis. CD8^+^ T cells were recruited more into APN KO BMSCs than into WT BMSCs (Fig. 7B). This result implies that APN KO BMSCs have a high ability to recruit CD8^+^ T cells to tumor tissues.

To determine which chemokines are responsible for the recruitment of CD8^+^ T cells into the tumor mass, the chemokine profiles were analyzed. CCL8 and *Ccl8* mRNA were increased in APN KO BMSCs (Fig. 7C, D) and CCL8 in cell culture supernatant were reduced by the treatment of the HIF1α inhibitor, vitexin (Fig. 7E). To clarify whether CCL8 from BMSCs is indeed responsible for the recruitment of CD8^+^ T cells into the tumor mass, splenocytes from EL-4-bearing mice were added to the upper chamber, and WT BMSCs or CCL8-added WT BMSCs were added to the bottom well at a ratio of 10:1. CD8^+^ T cells were more recruited into CCL8-added WT BMSCs than WT BMSCs (Fig. 7F). In addition, when CCL8-depleted APN KO BMSCs were placed in the bottom chamber, the recruitment of CD8^+^ T cells was decreased (Additional file 1: Figure S3C and S3D).

### Enhanced migration of BMSCs from old APN KO mice suppresses the growth of EL-4 tumors

**Fig. 8:**
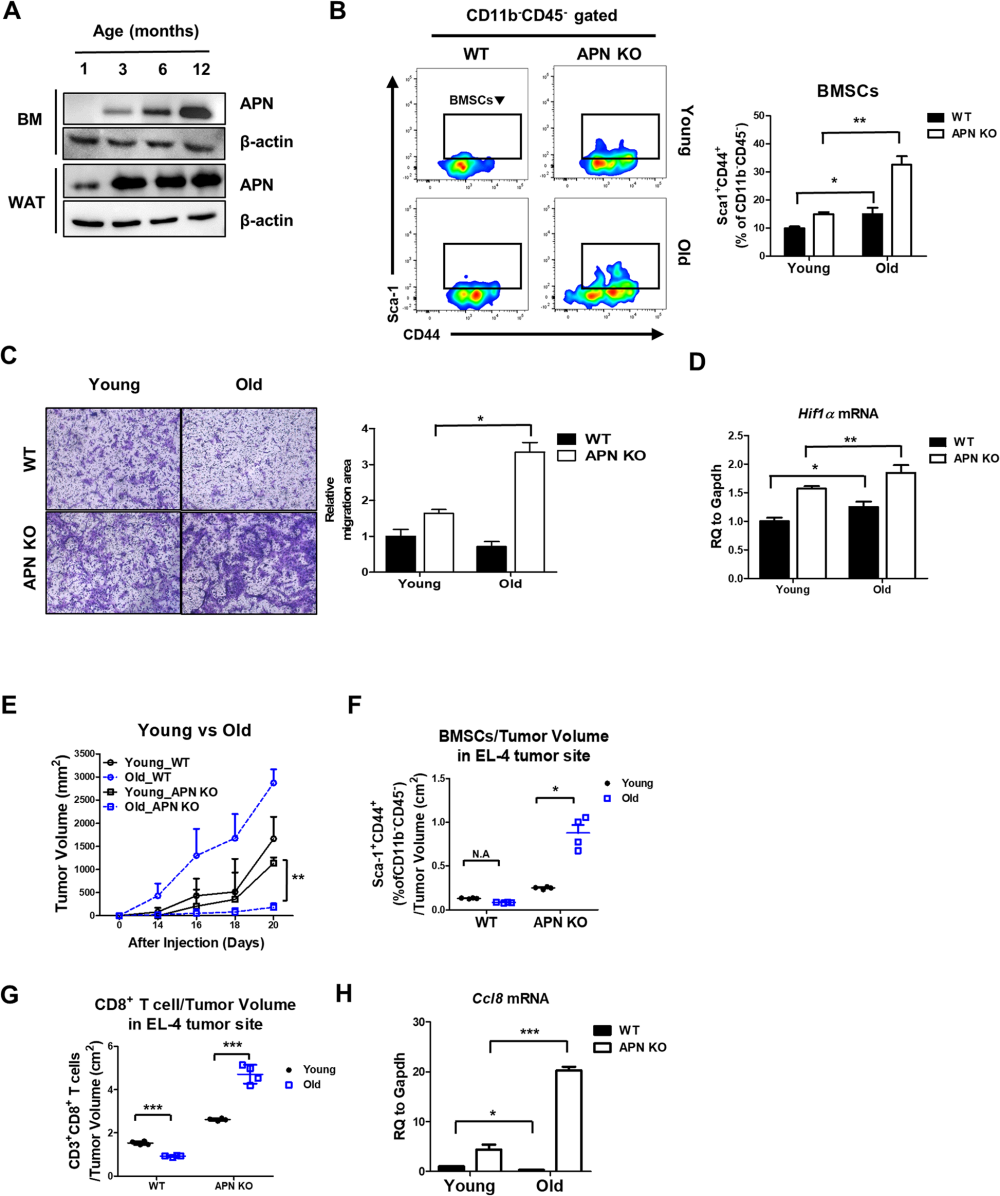
Old APN KO mice shows the most highly retarded EL-4 tumor growth via increase in infiltration of BMSCs and CD8^+^ T cells. **A** The protein levels of APN in both bone marrow (BM) and white adipose tissue (WAT) at indicated months were measured by western blotting. **B** The population of young and old APN KO BMSCs (Sca-1^+^CD44^+^CD11b^−^CD45^−^) cultured under 1% hypoxia was analyzed by flow cytometry, and bar graphs were used for quantitative data. **C** BMSCs were placed in the upper chamber of the transwell plate and migrated cells were imaged under light microscopy (× 100 magnification) after 24 h, and then images were quantified using ImageJ software. **D** The mRNA level of *HIF1α* in BMSCs from young and old APN KO mice was measured by real-time PCR **E** EL-4 tumors were excised from young and old APN KO mice 20 days after administration, and tumor volume was measured. **F** The population of infiltrated BMSCs (Sca-1^+^CD44^+^CD11b^−^CD45^−^) in EL-4 was analyzed by flow cytometry. **G** The population of infiltrated CD8^+^ T cells (CD3^+^CD8^+^) in EL-4 cells was analyzed by flow cytometry. **H** The mRNA level of *Ccl8* in young and old APN KO mice was measured by real-time PCR. All data were expressed as the mean ± SD from at least three independent experiments. *p < 0.05, **p < 0.01, ***p < 0.001

Although it is well-known that the BM is progressively filled with adipocytes from to 3–4 weeks after birth, little is known about what function of BMSCs become affected by APN in a long-term manner. Hence. we investigated the effect of increased APN on the BMSCs migration using aged mice. Since level of APN in BM began to increase from 3 to 4-month-old age, 3 to 4-month-old mice are defined as young mice and 11 to 12-month-old mice as old mice (Fig. 8A). To explore effect of APN in BMSCs in a long-term manner, we cultured young and old BMSCs from both WT and APN KO mice. We examined the difference of population and migration ability between young and old APN KO BMSCs. The population of BMSCs from old APN KO mice was twofold higher than that from young APN KO mice, while the population of old WT BMSCs was also marginally increased compared to that of young WT BMSCs (Fig. 8B). The migration of old APN KO BMSCs enhanced compared to young APN KO BMSCs and the Hif1α mRNA expression was also enhanced in old APN KO BMSCs compared to young APN KO BMSCs (Fig. 8C, D), implying that the increase in population and migration is likely due to HIF1α increase in old APN KO BMSCs. Next, the difference in EL-4 growth between young and old APN KO mice was also examined. Tumor growth was greatly retarded in old APN KO mice compared with that in young APN KO mice (Fig. 8E). Thus, we inferred that more BMSCs would be recruited to EL-4 tumor sites in old-aged APN KO mice. Indeed, the number of BMSCs per unit weight of EL-4 tumor tissue in old-aged APN KO mice was highly increased (Fig. 8F) and the CD8^+^ T cells/tumor weight ratio was increased about three-fold in old-aged APN KO mice compared with young-aged APN KO mice (Fig. 8G). Furthermore, the expression of *Ccl8* mRNA was higher than that of young APN KO BMSCs (Fig. 8H). These results address that APN may progressively reduce proliferation and migration ability of BMSCs.

## Discussion

Several previous reports have shown that APN has a positive regulatory role in the migration of some cell types. APN enhances the migration of endothelial progenitor cells (EPCs) mainly through the PI3-kinase signaling pathway [41], and the migration of Nestin^+^ BMSCs to the calvarial bone defect site via the bloodstream to regenerate the cells at the bone injury site [42]. In contrast, we found that APN depletion enhanced the migration of BMSCs under hypoxic conditions. This discrepancy could be explained by two possibilities. 1) APN KO BMSCs are influenced by APN deficiency environment for a longer time and were cultured in the hypoxic condition, whereas BMSCs cultured under normoxic conditions were used for the experiments [42, 43]. 2) we cultured BMSCs with MSC-specialized media under hypoxic conditions to enrich Sca1^+^CD44^+^ CD11b^−^CD45^−^BMSCs. This hypoxic culture condition to mimic the microenvironment of BM may allow the growth or differentiation of a unique subtype of BMSCs.

CD44 is a critical molecule that triggers the migration of BMSCs by cytoskeletal rearrangement [44]. When CD44 binds to HA, the RhoA/Rock/MLC signaling pathway is activated to increase actomyosin contractility in the CD44 expression cells [45, 46]. Interestingly, we found that CD44 expression in APN KO BMSCs was more enhanced than that in WT BMSCs under hypoxic conditions. Thus, CD44 expressing APN KO BMSCs highly moved toward HA-secreting EL-4 lymphoma compared to WT BMSCs. The CD44-HA interaction critically contributes to the migration of BMSCs toward EL-4 lymphoma in our experimental model. This molecular mechanism is able to be adapted to the clinical lymphoma model, because lymphoma shows high expression levels of HA [47], and patients with malignant lymphoma have clinically high serum levels of HA [48, 49]. On the other hand, as HAS3 under expression is associated with advanced tumor stage [50] and adverse pathological features in patients with urothelial carcinoma of upper urinary tract and urinary bladder [51], low BMSCs infiltration would be one of the causes if low infiltration of CD8^+^ T cells is a feature of urothelial carcinoma. Further studies of the correlation of CD44 expressing BMSCs and HAS3-expressing tumors may give us invaluable information.

It is known that hypoxic conditions increase the migration of BMSCs by enhancing the SDF-1/CXCR4 signaling pathway [28]. However, SDF-1/CXCR4 was unlikely to be the main molecular player because no significant difference in SDF-1 expression was observed between WT and APN KO BMSCs, although SDF-1 was significantly upregulated compared with other chemokines (Fig. 7C). Instead of activating the SDF-1/CXCR4 signaling pathway, we found that SCF-activated STAT3 phosphorylation enhanced HIF1α production and HIF1α also stimulates SCF. Thus, the SCF/STAT3/HIF1α positive feedback loop is likely to be activated in the absence of APN. Therefore, it is conceivable that APN plays a negative role in regulating the SCF/STAT3/HIF1α positive feedback loop on the migration of BMSCs. Indeed, APN activated GSK3β activity in BMSCs (Fig. 4B) and the GSK3β inhibitor promoted the stabilization of HIF1α (Fig. 4D). Thus, it is proven that APN acts as a negative regulator of HIF1α-mediated BMSCs migration.

We previously showed that EL-4 lymphoma growth was retarded by increased CTLs and NK cells in APN KO mice [29]. In this study, we demonstrated that APN KO BMSCs highly migrated into the EL-4 tumor mass and secreted CCL8, resulting in the recruitment of CD8^+^ T cells. This result provides a more detailed mechanism by which EL-4 tumor mass growth is retarded in APN KO mice. On the other hand, in case of APN KO mice, EL-4 tumor growth in old APN KO mice was more retarded than in young APN KO mice (Fig. 8E) and CD8^+^ T cells per tumor weight dramatically increased in old-aged APN KO mice compared with young-aged APN KO mice (Fig. 8G). This is the underlying mechanism why EL-4 tumor growth is slower in old-aged APN KO mice than in young-aged APN KO mice. On the other hand, it is reported that APN does not directly affect the migration of CD8^+^ T cells since the level of APN receptors on T cells is low [52]. However, our study demonstrates that APN indirectly stimulates the migration of CD8^+^ T cells via CCL8 expressing BMSCs.

MSCs are the origin of BMAs as in osteoblasts and BMAs are progressively filled in BM environment along with the age [53, 54], reached to around 10% of the total adipose mass [8]. These unique BMAs are derived from CD150^−^CD48^−^Lineage^−^Sca-1^+^c-kit^+^ [55, 56] or LepR^+^CD45^−^Ter119^−^CD31^−^MSCs [57] in the BM and regulate systemic metabolism as important endocrine cells and have both positive and negative effects on bone density and hematopoiesis [58]. Indeed, the APN secreted from BMAs stimulates the differentiation of resident BMSCs toward fat rather than bone [59], leading to the increased risk of osteoporosis along with the age. Thus, it is also intriguing to question how APN affects the other functions of BMSCs according to the age. For instance, APN signaling stimulates pro-osteogenic and anti‐osteoclastogenic potential of MSCs in young mice [60]. APN receptor activation improves age-related skeletal muscle dysfunction via the stimulation of aged muscle satellite cells, but shows contrary effect in young muscle satellite cells [61].

**Fig. 9:**
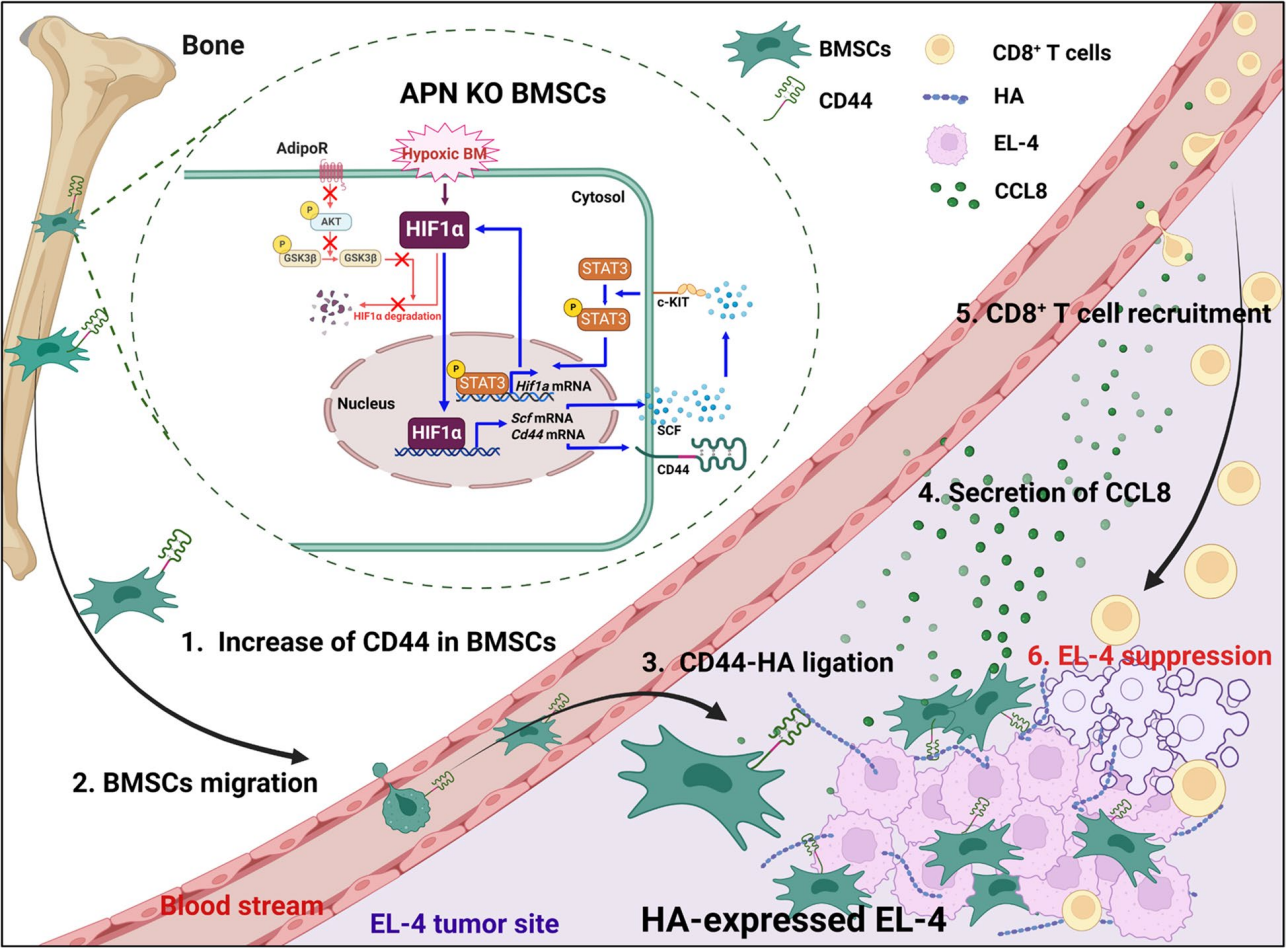
Graphic summary. In the absence of APN, hypoxia stabilized HIF1α increases SCF production. Secreted SCF activates STAT3, which is involved in HIF1α transcription (blue line arrow). In the presence of APN, HIF1α is degraded via activation of the AdipoR/GSK3β axis, which prevents amplification of the HIF1α signaling pathway (red line arrow). This implies that APN acts as a breaker in HIF1α amplification. On the other hand, HIF1α stimulates CD44 expression, and CD44-expressing BMSCs move into an HA-rich EL-4 tumor mass, in which BMSCs secrete CCL8 to recruit CD8^+^ T cells. This pathway contributes to the inhibition of tumor growth

In the present study, we revealed that the migration ability of old-aged APN KO BMSCs was increased in the syngeneic tumor model and that the migrated BMSCs recruited more CD8^+^ T cells compared with young-aged APN KO BMSCs, which results in the retardation of tumor growth. The summary of our study is illustrated in Fig. 9.

## Conclusion

In conclusion, we reveal that the migration ability of APN KO BMSCs is enhanced by the stimulation of SCF/STAT3/HIF1α axis under hypoxic conditions similar to the BM niche. Thus, APN KO BMSCs highly migrate toward EL-4 tumor mass by CD44-HA interaction and recruit CD8^+^ T cells by releasing of CCL8. It implies that APN regulates the function of BMSCs related to CD44-mediated migration. In addition, this accumulative effect of APN deficiency on EL-4 tumor growth is likely to be increased along with age. Now, age-related other function of APN in BM environment is worth elucidating and little attention to BMAs is changing thanks to technical improvement to solve these questions.

### Supplementary Information

**Additional file 1: Figure S1.** A The scheme of the Hif1α promoter (P) region with the location of the putative STAT3 binding sites. **Figure S2.** A The level of *Has3* mRNA was measured using real-time PCR. The data are expressed as mean ± SD from at least three independent experiments. *p < 0.05, ***p < 0.001. **Figure S3.** A Representative immunofluorescence IHC staining images were used to detect CD8^+^ and Sca-1^+^ from EL-4 tumor sections and shown as a quantitative bar graph. Scale bar = 300 μm. B The population of splenic CD8^+^ T cells (CD3^+^CD8^+^) in EL-4 bearing mice was analyzed by flow cytometry. The bar graphs are used for quantitative data. C The mRNA level of *Ccl8* in BMSCs transfected with CCL8 siRNA was measured by real-time PCR. D WT and APN KO BMSCs were placed in the bottom chamber of the transwell plate with CCL8 siRNA treatment. Splenocytes from EL-4-bearing WT mice were placed in the upper chamber of the transwell plate and migrated CD8^+^ T cells in the bottom chamber were analyzed by flow cytometry. All data are expressed as means ± SD from at least three independent experiments. *p < 0.05, **p < 0.01, ***p < 0.001.

**Additional file 2: Table S1.** List of primers for RT-PCR. **Table S2.** List of primers for real-time PCR. **Table S3.** List of primers for CHIP-qPCR. **Table S4.** List of CCL8 specific siRNA sequences.

**Additional file 3.**

## Data Availability

The data that support the findings of this study are available from the corresponding author upon reasonable request.

## Abbreviations

AKT: Protein kinase B
AMPK: AMP-activated protein kinase
APN: Adiponectin
BM: Bone marrow
BMSCs: Bone marrow-derived mesenchymal stem cells
CCL8: Chemokine (C–C motif) ligand 8
CFU-F: Colony-forming unit-fibroblast
CXCR4: C-X-C chemokine receptor type 4
GAPDH: Glyceraldehyde 3-phosphate dehydrogenase
GSK3β: Glycogen synthase kinase 3beta
HA: Hyaluronic acid
HAS3: Hyaluronan synthase 3
HIF1α: Hypoxia inducible factor 1α
HSCs: Hematopoietic stem cells
IFN-γ: Interferon gamma
IL-1β: Interleukin 1 beta
IL-10: Interleukin 8
IL-6: Interleukin 6
KO: Knockout
MLC2: Myosin light chain 2
mRNA: Messenger RNA
MSCs: Mesenchymal stem cells
MYLK: Myosin light chain kinase
ROS: Reactive oxygen species
RQ: Relative quantification
RhoA: Ras homolog family member A
RT-PCR: Reverse transcription polymerase chain reaction
Rock1: Rho Associated Coiled-Coil Containing Protein Kinase 1
rAPN: Recombinant adiponectin
Sca-1: Stem cells antigen-1
SCF: Stem cell factor
SDF-1: Stromal cell-derived factor-1
STAT3: Signal transducers and activators of transcription
TME: Tumor microenvironment
TNF-α: Tumor necrosis factor alpha
WT: Wild-type
4-Mu: 4-Methylumbelliferone
