# Correction: Adiponectin affects the migration ability of bone marrow-derived mesenchymal stem cells via the regulation of hypoxia inducible factor 1α

**DOI:** 10.1186/s12964-023-01248-4

**Published:** 2023-08-03

**Authors:** Sujung Soh, Sora Han, Hye In Ka, Se Hwan Mun, Woojung Kim, Gaeun Oh, Young Yang

**Affiliations:** 1 Department of Biological Sciences, Sookmyung Women’s University, Seoul, 04310 Republic of Korea; 2 Research Institute of Women’s Health, Sookmyung Women’s University, Seoul, 04310 Republic of Korea

**Correction: Cell Commun Signal 21, 158 (2023)**

**https://doi.org/10.1186/s12964-023-01143-y**

Following the publication of the original article [1], the authors found an error in Fig. 7B. Unintentionally, the wrong flow cytometry image was displayed in recruited CD8^+^ T cells toward APN KO BMSCs. To further verify the study's accuracy, we would like to provide the revised Fig. 7B. The correct image is presented in this correction article, and the correction does not change the description or the conclusion of the original paper.

**Fig. 7:**
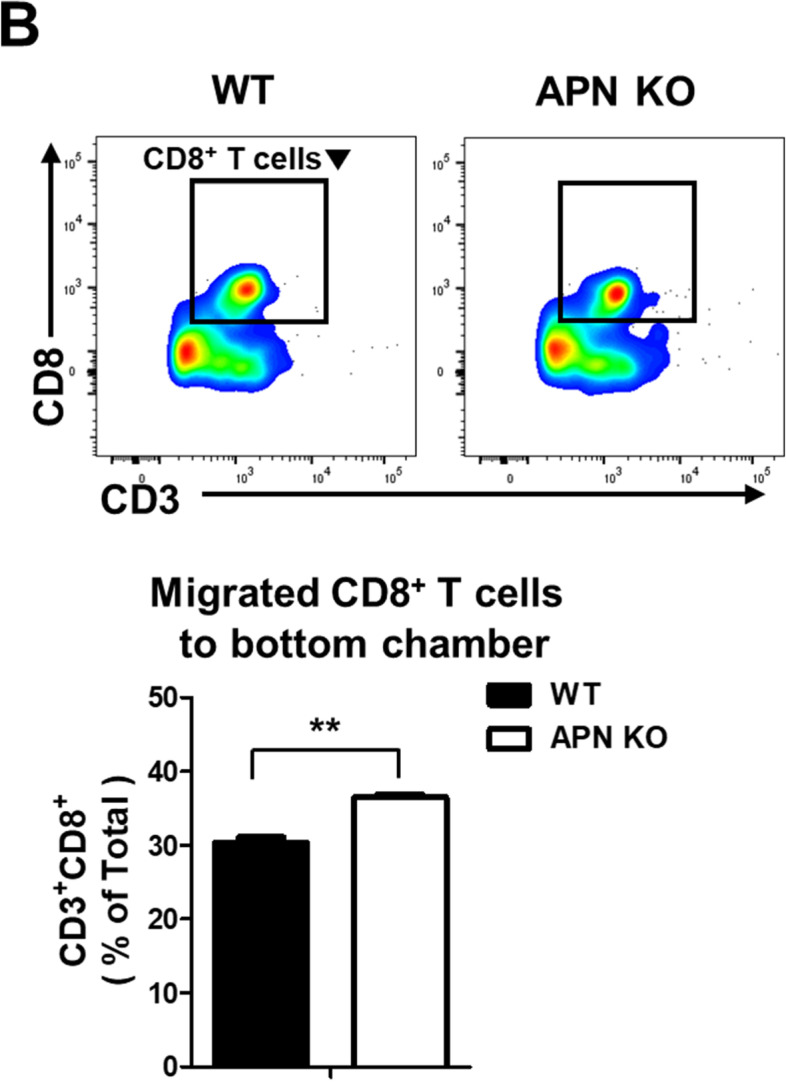
**B** APN KO BMSCs were placed in the bottom chamber, splenocytes from EL-4-bearing WT mice were placed in the upper chamber of the transwell plate, and the population of migrated CD8^+^ T cells in the bottom chamber was analyzed by flow cytometry. **C** Cell lysates from the BMSCs were used to measure the levels of various chemokines. All dots were quantified using ImageJ software, and bar graphs were used for quantitative data

Further to this, the discussion has been updated to: “Indeed, APN activated GSK3β activity in BMSC (Fig. 4B), and the GSK3β inhibitor promoted the stabilization of HIF1α (Fig. 4D).” We offer the revised sentence to clarify the content of the research article.

The authors would like to apologize for any inconvenience caused. The original article [1] has been corrected.
