# Supplementary figures and images for: Adiponectin affects the migration ability of bone marrow-derived mesenchymal stem cells via the regulation of hypoxia inducible factor 1α
Source: Cell Commun Signal. 2023 Jun 27;21:158. doi: 10.1186/s12964-023-01143-y (PMC10294307; doi:10.1186/s12964-023-01143-y)

Additional file 1: Figure S1

A

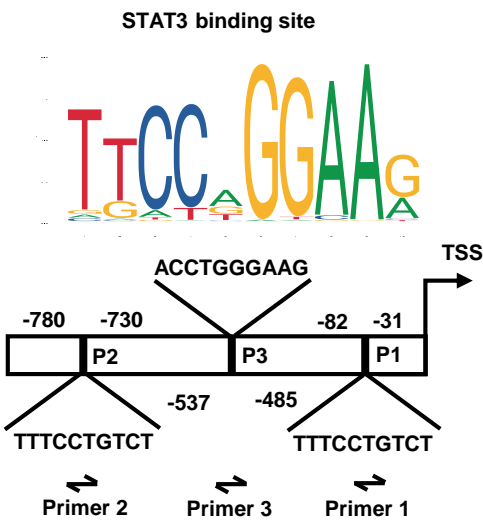

Additional file 1: Figure S2

A

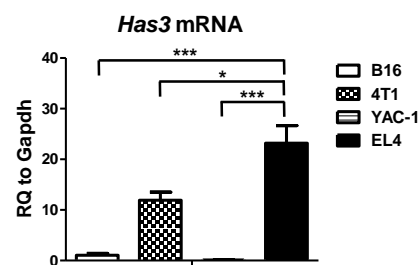

Additional file 1: Figure S3

A

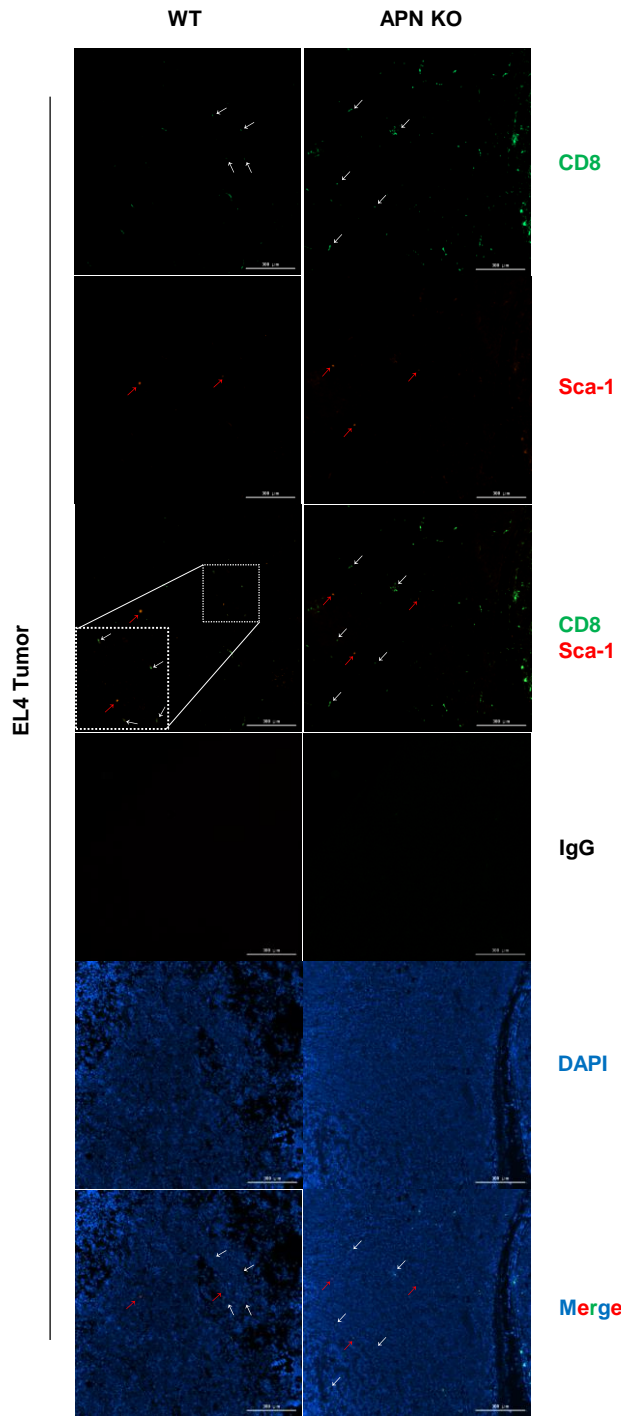

B

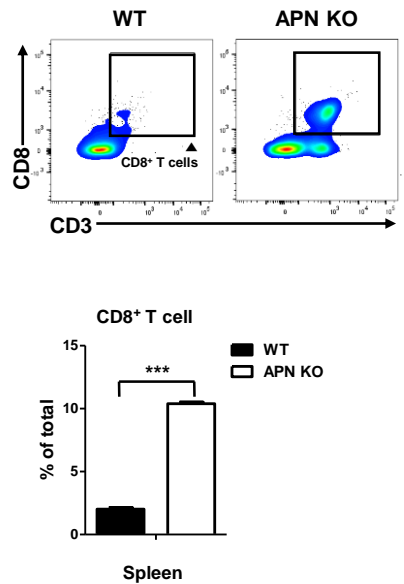

C

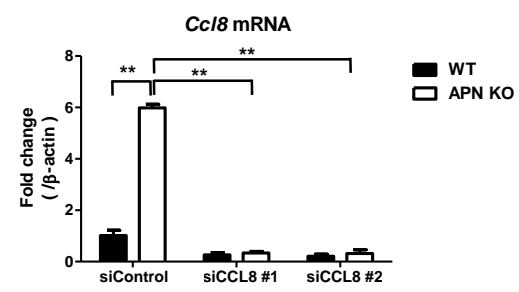

D

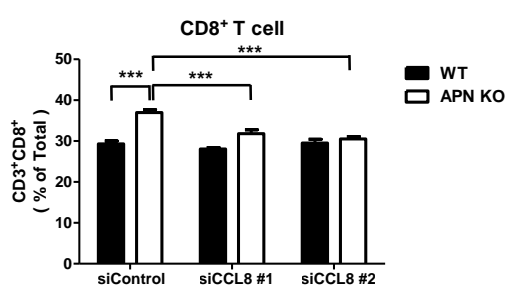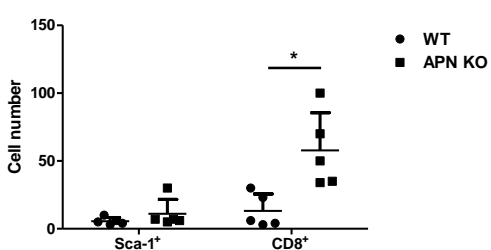

Supplement: Supplementary file 2 — Additional file 1: Figure S1. A The scheme of the Hif1α promoter (P) region with the location of the putative STAT3 binding sites. Figure S2. A The level of Has3 mRNA was measured using real-time PCR. The data are expressed as mean ± SD from at least three independent experiments. *p < 0.05, ***p < 0.001. Figure S3. A Representative immunofluorescence IHC staining images were used to detect CD8+ and Sca-1+ from EL-4 tumor sections and shown as a quantitative bar graph. Scale bar = 300 μm. B The population of splenic CD8+ T cells (CD3+CD8+) in EL-4 bearing mice was analyzed by flow cytometry. The bar graphs are used for quantitative data. C The mRNA level of Ccl8 in BMSCs transfected with CCL8 siRNA was measured by real-time PCR. D WT and APN KO BMSCs were placed in the bottom chamber of the transwell plate with CCL8 siRNA treatment. Splenocytes from EL-4-bearing WT mice were placed in the upper chamber of the transwell plate and migrated CD8+ T cells in the bottom chamber were analyzed by flow cytometry. All data are expressed as means ± SD from at least three independent experiments. *p < 0.05, **p < 0.01, ***p < 0.001. [file 12964_2023_1143_MOESM1_ESM.pdf]
