# Supplementary material for: Adiponectin affects the migration ability of bone marrow-derived mesenchymal stem cells via the regulation of hypoxia inducible factor 1α
Source: Cell Commun Signal. 2023 Jun 27;21:158. doi: 10.1186/s12964-023-01143-y (PMC10294307; doi:10.1186/s12964-023-01143-y)
Supplement: Supplementary file 3 — Additional file 2: Table S1. List of primers for RT-PCR. Table S2. List of primers for real-time PCR. Table S3. List of primers for CHIP-qPCR. Table S4. List of CCL8 specific siRNA sequences. [file 12964_2023_1143_MOESM2_ESM.pdf]

Additional file 2: Table S1

Gene	bp	Forward	Reverse
Hif1a	561	AGG ATG AGT TCT GAA CGT CGA AA	AAT ATG GCC CGT GCA GTG AA
Ccl8	220	CTG CTC ATA GCT GTC CCT GTC	ACT CAC TGA CCC ACT TCT GTG
Sdf-1	608	TGT TGC CAT GGA ACC GAT CA	TCC ACA GGC TAT CGG GGT AA
Ccl12	298	TATTGGCTGGACCAGATGCG	GGGGAACTTCAGGGGGAAAT
Ccl6	196	GGC TGG CCT CAT ACA AGA AA	TCC CCT CCT GCT GAT AAA GA
Mip1-γ	300	CAG GCC GGG CAT CAT CTT TA	GGC ACA GAC AAG TTT CCC CT
Chemerin	307	AAG GCC TCG CTA AAG CAA CA	CTT CTT GGG GCA GTT GGT CT
β-actin	540	GTGGGCCGCCCTAGGCACCA	CTCTTTGATGTCACGCACGA

Additional file 2: Table S2

Gene	bp	Forward	Reverse
Il-6	116	TAC CAC TTC ACA AGT CGG AGG C	CTG CAA GTG CAT CAT CGT TGTTC
Sox2	138	AAC GGC AGC TAC AGC ATG ATG C	CGA GCT GGT CAT GGA GTT GTA C
Has3	129	CCT TGG CAA CTC AGT GGA CTA C	TGG ACA TCT CCT CCA ACA CCT C
Nanog	141	GAA CGC CTC ATC AAT GCC TGC A	GAA TCA GGG CTG CCT TGA AGA
Hif1α	110	CCT GCA CTG AAT CAA GAG GTT GC	CCA TCA GAA GGA CTT GCT GGC T
Ccl8	122	TCT ACG CAG TGC TTC TTT GCC	AAG GGG GAT CTT CAG CTT TAG TA
Ccl6	196	GGC TGG CCT CAT ACA AGA AA	TCC CCT CCT GCT GAT AAA GA
Tnf-α	139	GGT GCC TAT GTC TCA GCC TCT T	GCC ATA GAA CTG ATG AGA GGG AG
Scf	65	CCC TGA AGA CTC GGG CCT A	CAA TTA CAA GCG AAA TGA GAG CC
Lif	244	CAA GAA TCA ACT GGC ACA GC	AGT GGG GTT CAG GAC CTT CT
Il-1β	240	AGG AGA ACC AAG CAA CGA CA	TCT GCT TGT GAG GTG CTG AT
Oct4	160	TAGGTGAGCCGTCTTTCCAC	GCTTAGCCAGGTTCGAGGAT
Gapdh	125	GTG GAC CTC ATG GCC TAC AT	TGT GAG GGA GAT GCT CAG TG
Ifn-r	165	TTCTTCAGCAACAGCAAGGC	TCAGCAGCGACTCCTTTTCC
Il-10	71	GCC ACA TGC TCC TAG AGC TG	CAG CTG GTC CTT TGT TTG AAA

Additional file 2: Table S3

	bp	Forward	Reverse
**Primer 1**	51	GCC TGC GTC CTT CAC CAT	TCA CCT GAG CGG AGG C
**Primer 2**	50	TTC AAT GCC TTT TCC TGT CTT GTC	ATA AGT GAG AGC ACA AGC CTG A
**Primer 3**	52	CTG GAG ACT GAG TGG TGT CG	TGT TAG AGT GGG GTC CCT CC

Additional file 2: Table S4

	Sequences
**siCCL8 #1**	GAG AUG UUA CAA GCA GAU Gtt
**siCCL8 #2**	CUG CAG CCU UGA ACC UUC Att
