# Supplementary material for: Adiponectin affects the migration ability of bone marrow-derived mesenchymal stem cells via the regulation of hypoxia inducible factor 1α
Source: Cell Commun Signal. 2023 Jun 27;21:158. doi: 10.1186/s12964-023-01143-y (PMC10294307; doi:10.1186/s12964-023-01143-y)

Uncropped data_Figure 2

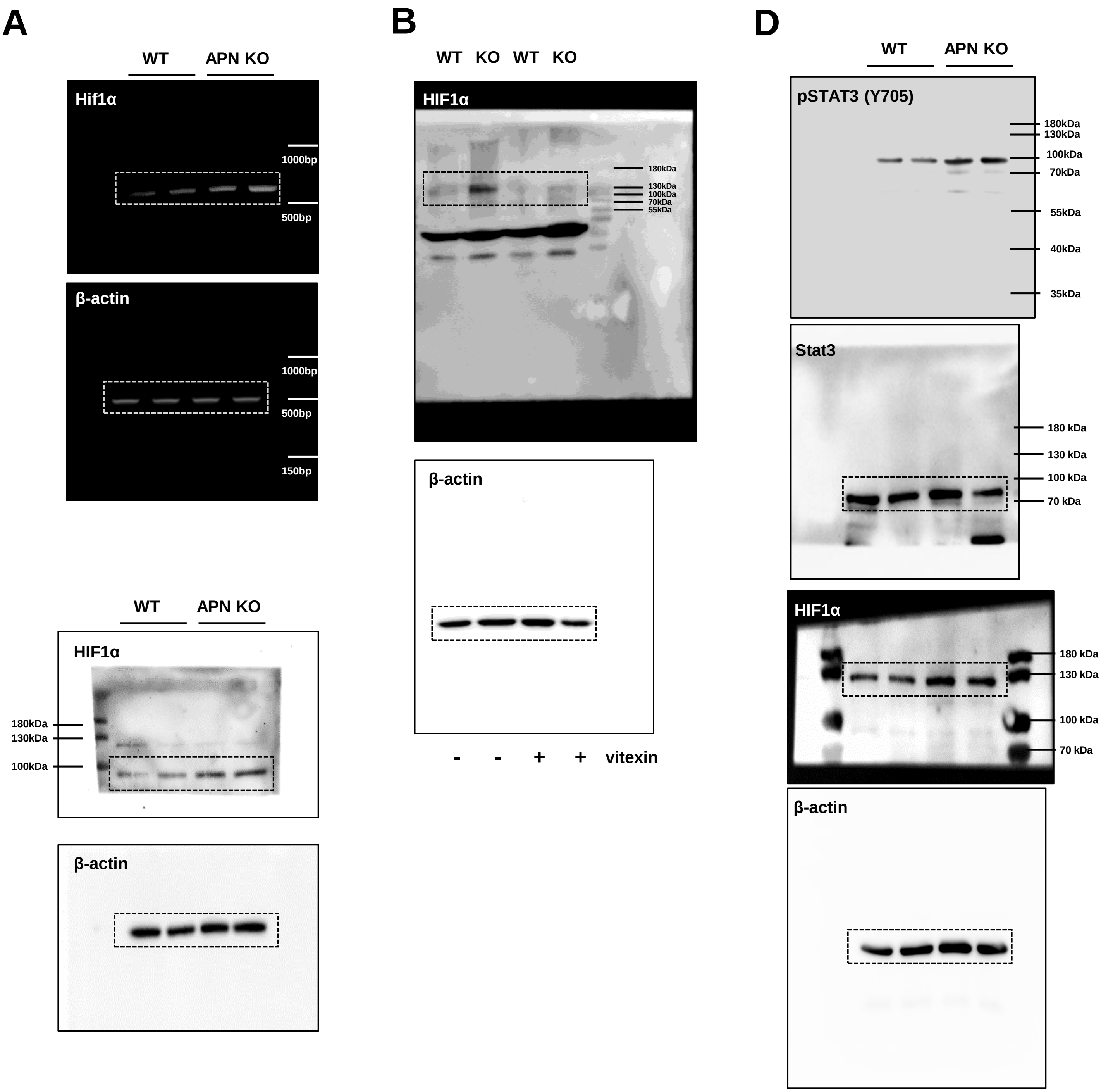

Uncropped data_Figure 2

E

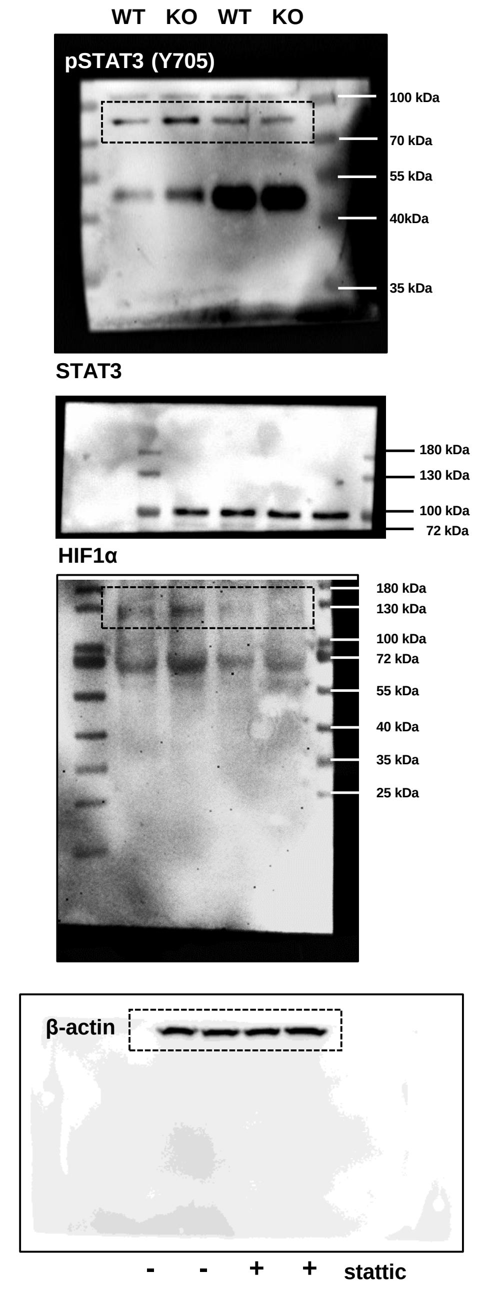

Uncropped data_Figure 3

C

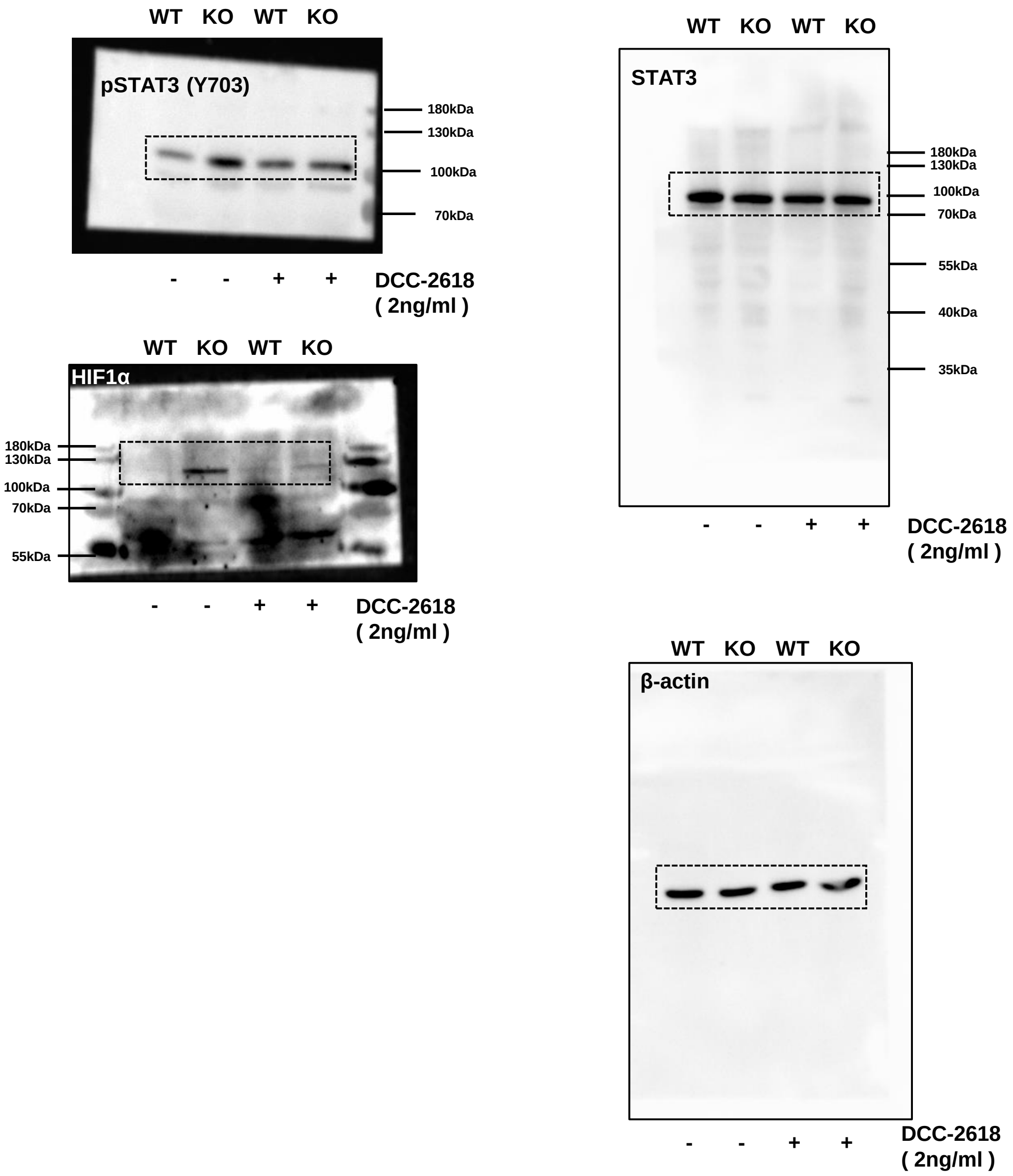

Uncropped data_Figure 4

A

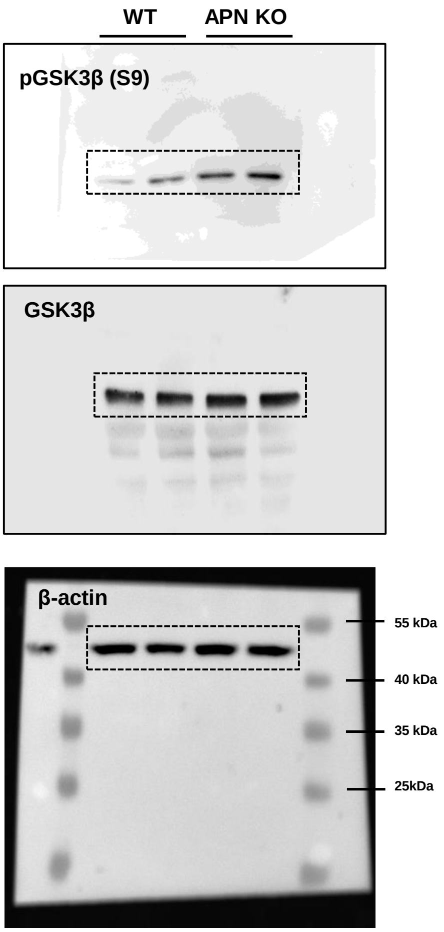

B

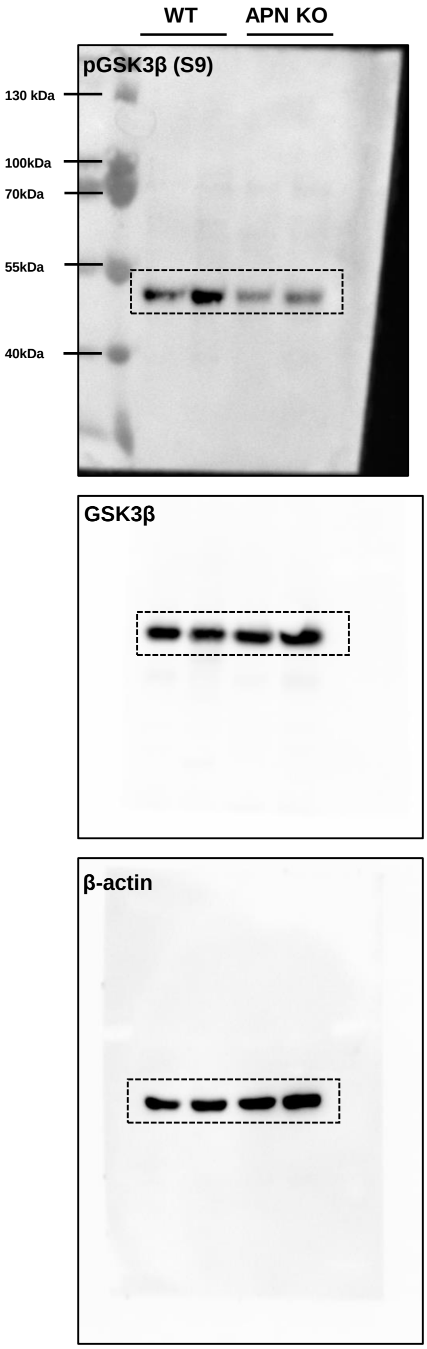

Uncropped data_Figure 4

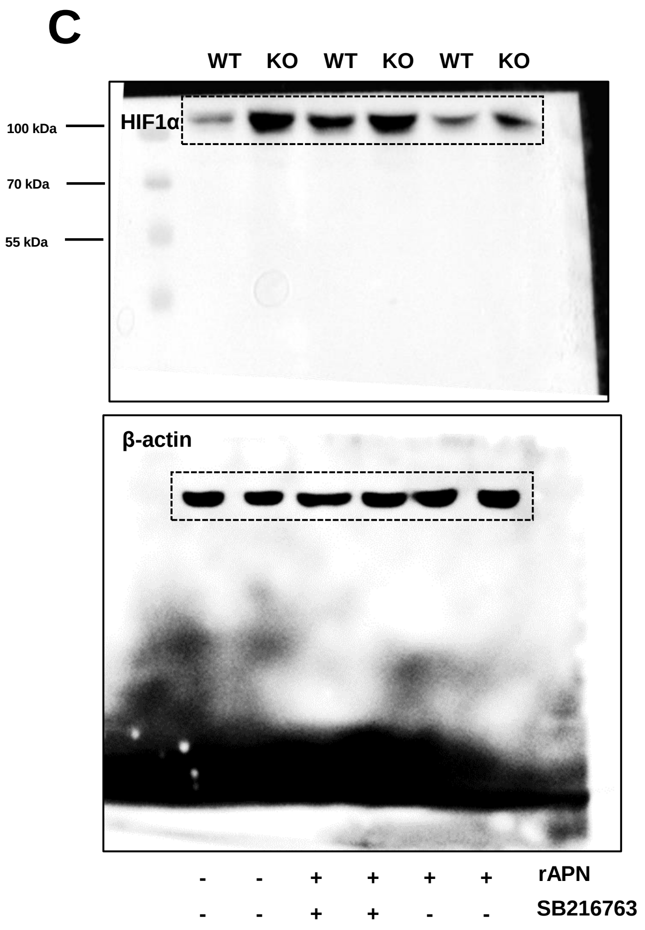

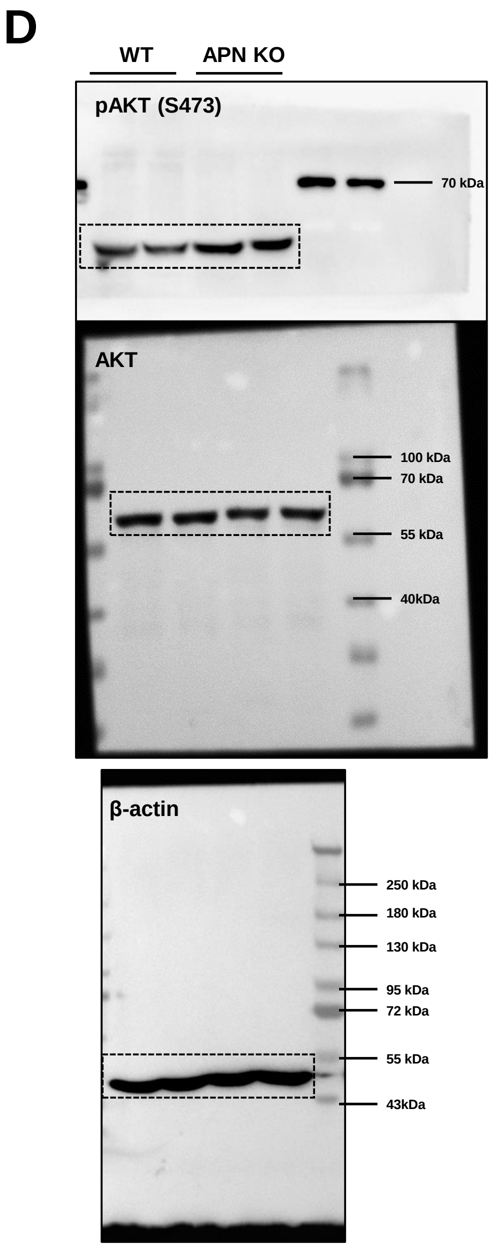

Uncropped data_Figure 5

E

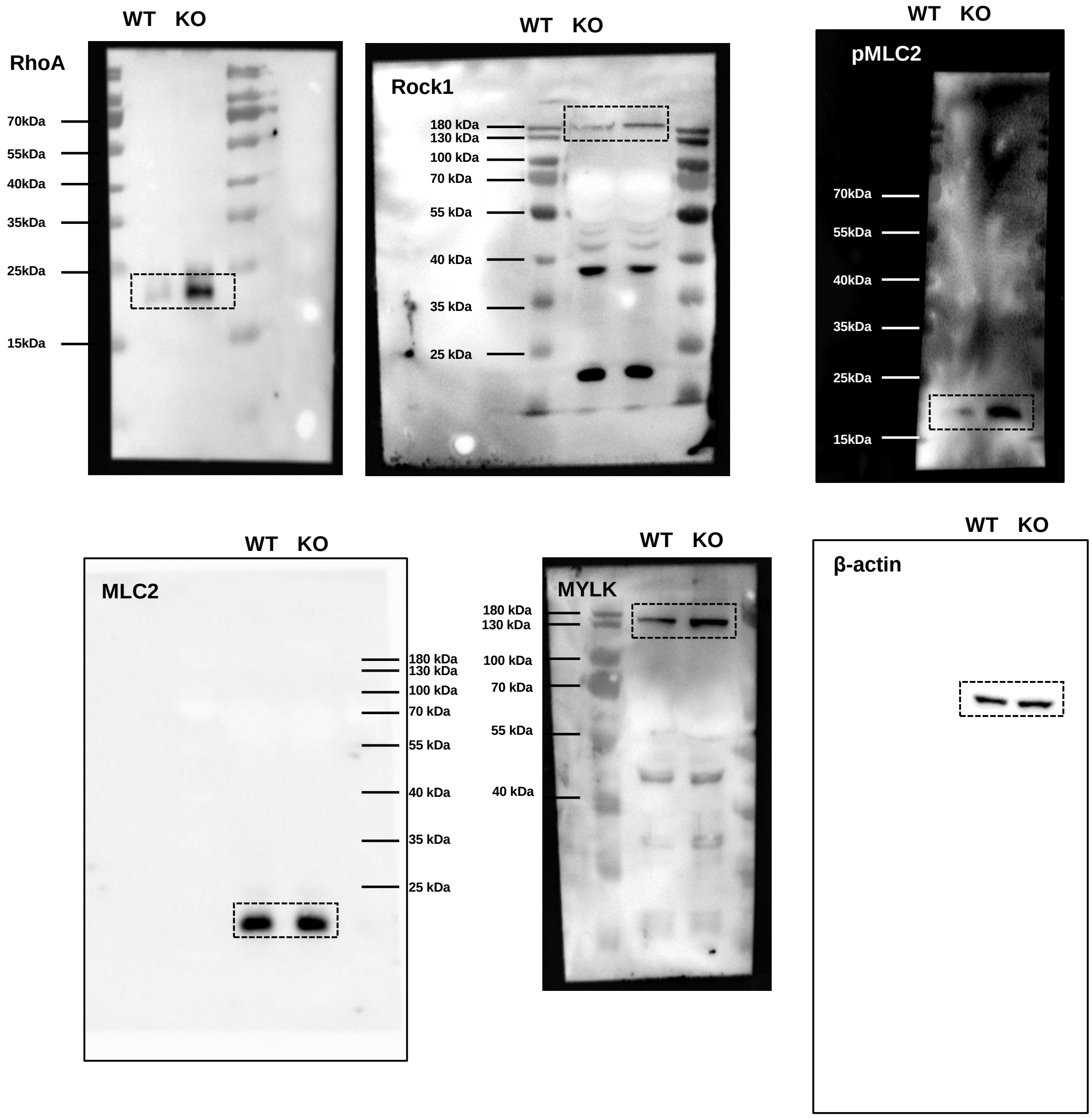

Uncropped data_Figure 7

C

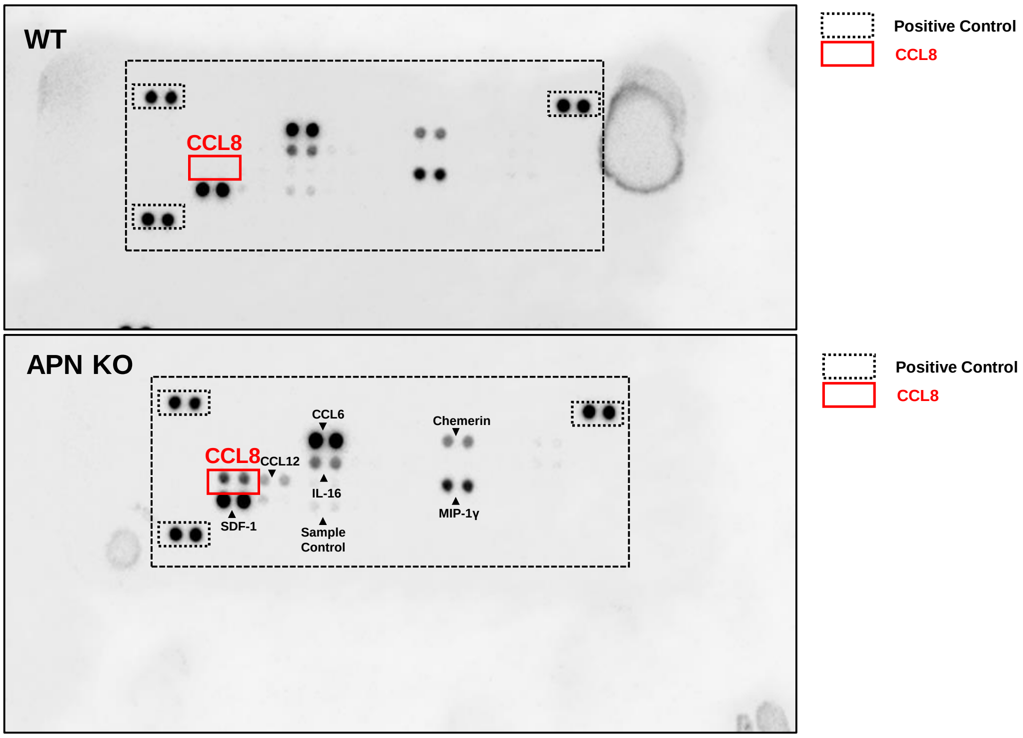

D

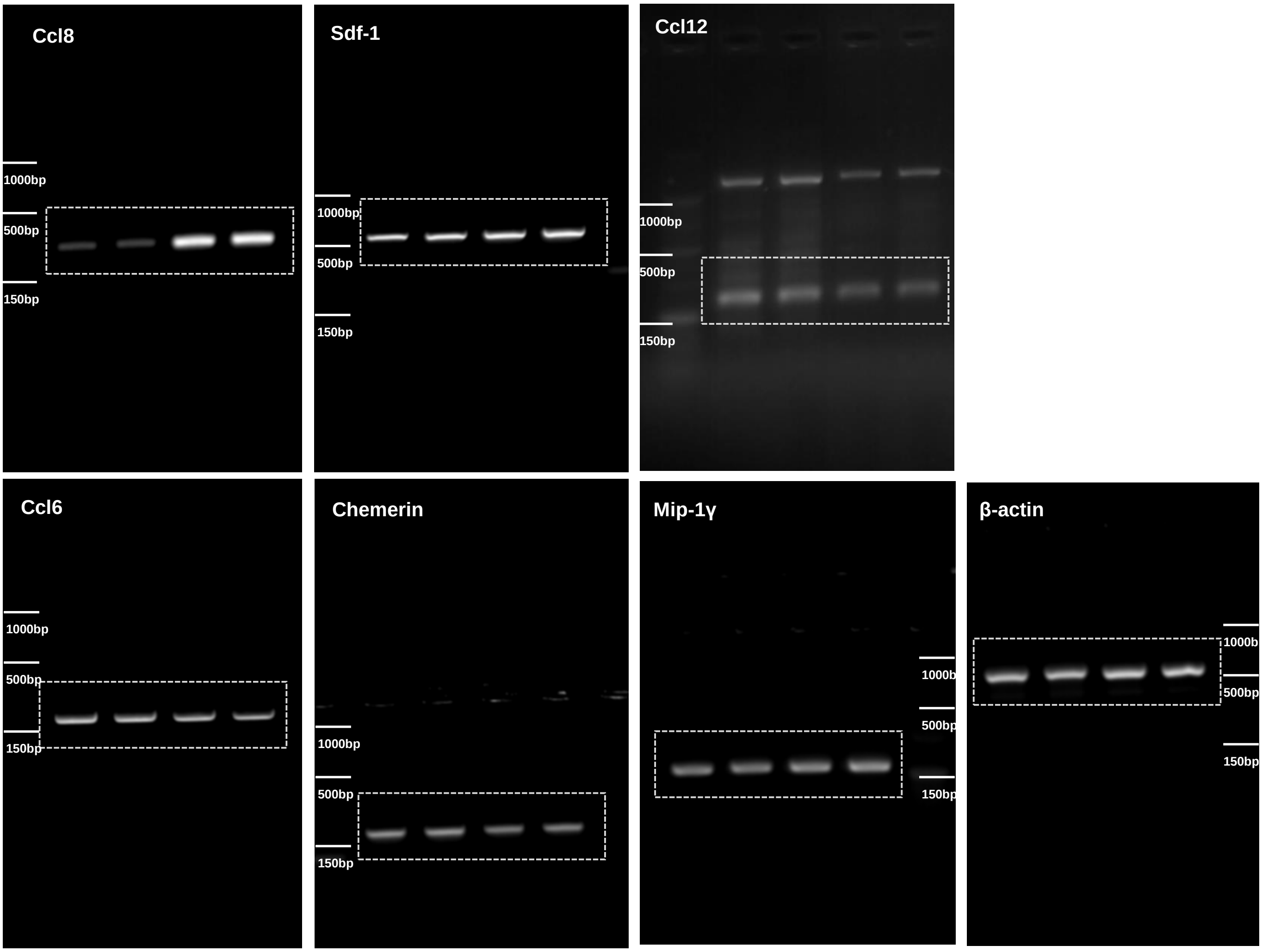

Uncropped data_Figure 8

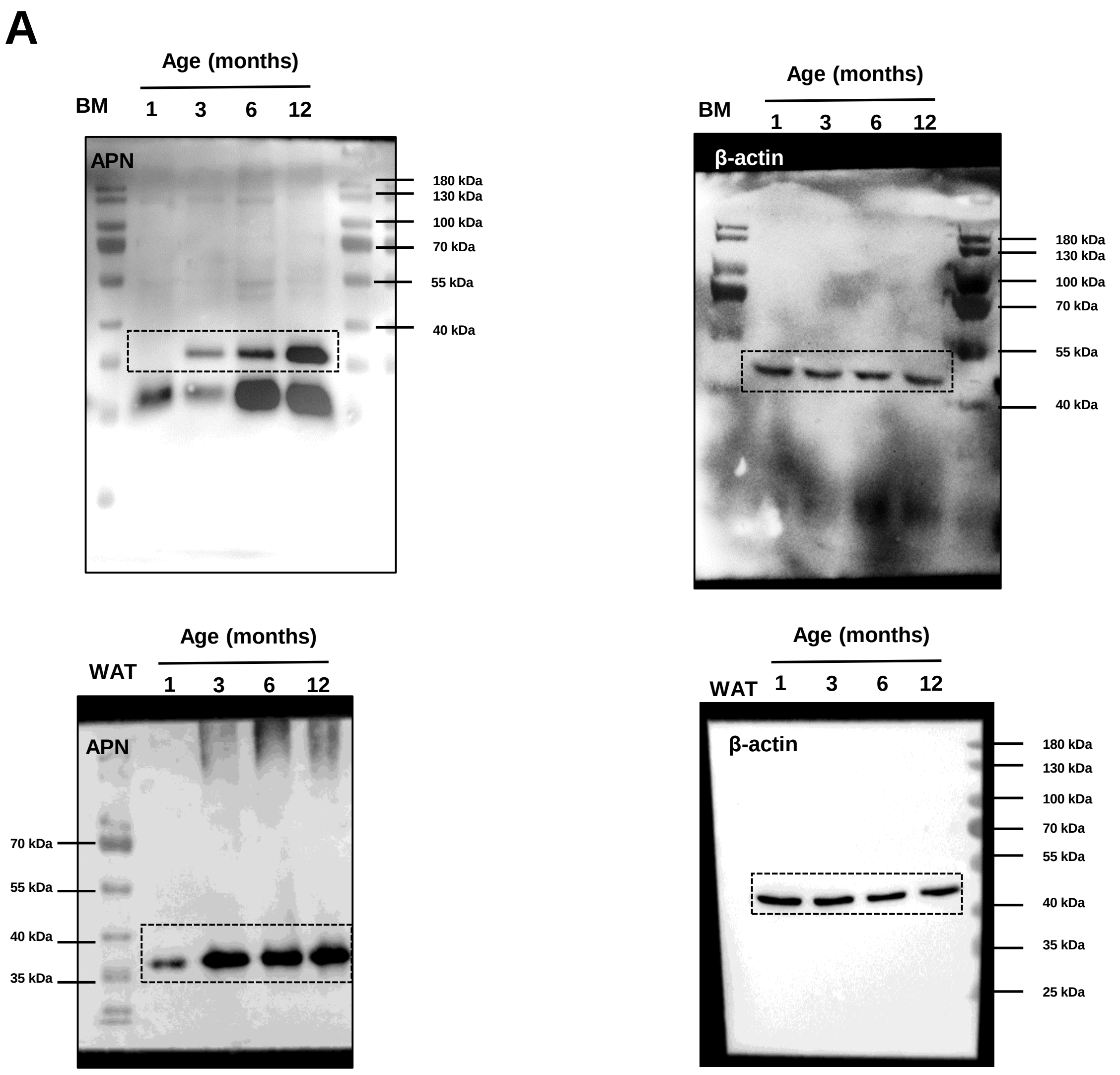

Supplement: Supplementary file 4 — Additional file 3. [file 12964_2023_1143_MOESM3_ESM.pdf]
